# Looking for immediate and downstream evidence of lexical prediction in eye movements during reading

**DOI:** 10.1177/17470218231223858

**Published:** 2024-01-27

**Authors:** Roslyn Wong, Aaron Veldre, Sally Andrews

**Affiliations:** 1 School of Psychology, The University of Sydney, Sydney, New South Wales, Australia; 2 School of Psychological Sciences, Macquarie University, Sydney, New South Wales, Australia

**Keywords:** Reading, eye movements, predictability effects, prediction cost, downstream processing

## Abstract

Previous investigations of whether readers make predictions about the full identity of upcoming words have focused on the extent to which there are processing consequences when readers encounter linguistic input that is incompatible with their expectations. To date, eye-movement studies have revealed inconsistent evidence of the processing costs that would be expected to accompany lexical prediction. This study investigated whether readers’ lexical predictions were observable during or downstream from their initial point of activation. Three experiments assessed readers’ eye movements to predictable and unpredictable words, and then to subsequent downstream words, which probed the lingering activation of previously expected words. The results showed novel evidence of processing costs for unexpected input but only when supported by a plausible linguistic environment, suggesting that readers could strategically modulate their predictive processing. However, there was limited evidence that their lexical predictions affected downstream processing. The implications of these findings for understanding the role of prediction in language processing are discussed.

It is well established that a word’s predictability, as indexed by cloze probability (i.e., the proportion of individuals that provide a given word for an unfinished sentence frame in an offline task; Taylor, 1953), is an important factor that determines how readers process words within a sentence. Early behavioural studies using naming (Stanovich & West, 1983; Traxler & Foss, 2000) and lexical decision tasks (Fischler & Bloom, 1985; Kleiman, 1980; Schwanenflugel & LaCount, 1988; Schwanenflugel & Shoben, 1985) have demonstrated that predictable words elicit decreased response times compared with unpredictable words. Similarly, eye-movement studies of sentence reading have found that predictable words are more likely to be skipped and to receive shorter fixation durations compared with unpredictable words (Balota et al., 1985; Drieghe et al., 2005; Ehrlich & Rayner, 1981; Frisson et al., 2017; Luke & Christianson, 2016; Rayner et al., 2011; see Staub, 2015 for a review). Research using event-related potentials (ERPs) has also revealed a graded relationship between word predictability and the N400 component, a centro-parietal negativity that peaks around 300 to 500 ms post-stimulus onset. Predictable words consistently yield smaller N400 components than unpredictable words, which is thought to reflect the ease of semantic processing (Federmeier et al., 2007; Kutas et al., 1984; Kutas & Hillyard, 1984; Thornhill & Van Petten, 2012). Thus, there is consistent evidence across different methodologies that words that can be predicted from prior context are processed more efficiently during online language comprehension (see Kuperberg & Jaeger, 2016 for a review).

Despite the extensive research into predictability effects, there is still considerable debate about whether these facilitatory effects are the result of anticipatory prediction—the “all-or-none process of activating . . . a word in advance of perceptual input” (DeLong, Troyer, & Kutas, 2014, p. 632). Although anticipatory prediction is assumed to ease the burden of processing noisy and informationally dense language input (Clark, 2013; Friston, 2010; Kutas et al., 2011), researchers have traditionally argued against a role for prediction during online processing. First, predictability effects have been proposed to reflect processes of post-lexical integration rather than prediction, i.e., a predictable word is easier to process, not because it has been preactivated ahead of time but because other linguistic information has been activated as a result of processing the input, which makes it easier to integrate into an unfolding discourse representation (Pickering & Gambi, 2018). Although prediction is difficult to disentangle from integration because both processes entail facilitated processing for predictable words (Kutas et al., 2011; but see Van Berkum et al., 2005; Wicha, Bates, et al., 2003; Wicha et al., 2004; Wicha, Moreno, & Kutas, 2003), predictability effects are typically observed on early eye-movement measures including skipping (Balota et al., 1985; Rayner et al., 2011), which would appear to be incompatible with post-lexical integration processes (Abbott & Staub, 2015; but see Veldre et al., 2020). Second, even if prediction does play a genuine part during online processing, very few words are predictable in natural language (Gough, 1983; Gough et al., 1981; Luke & Christianson, 2016; but see Cevoli et al., 2022)—the unconstrained nature of language means that infinite options are available as plausible continuations for each word of an unfolding sentence (Jackendoff, 2002). If this is the case, prediction would have limited utility for language comprehension beyond highly constraining, “prediction-friendly” contexts (Huettig & Mani, 2016). Finally, it remains unclear exactly what readers predict—although readers appear to preactivate morphosyntactic, syntactic, and semantic information during online processing (Luke & Christianson, 2016), there is inconsistent evidence that readers routinely make predictions about the full identity of upcoming words.

If readers make lexical predictions about upcoming words, there should be evidence of processing consequences when the anticipated input does not eventuate. For example, consider a strongly constraining sentence frame like “*The children went outside to . . .* ” for which most readers will expect the predictable completion “*play*.” If this prediction were to be disconfirmed by a plausible but unexpected completion like “*look*,” a prediction error cost should occur due to the mismatch between the word preactivated by the context and the input eventually encountered. However, the same, equally unpredictable completion “*look*” should not elicit a similar processing cost in a weakly constraining sentence frame like “*Joy was frightened to . . .* ” for which readers are unlikely to have made any predictions in advance of the upcoming text. Thus, the appearance of processing costs for unexpected input in strongly but not weakly constraining contexts should provide strong evidence that readers have made a predictive commitment to a specific lexical item.

Across studies and methodologies, however, there are notable inconsistencies about whether disconfirmed predictions give rise to the processing costs that would be expected to accompany lexical prediction. For example, Frisson et al. (2017) presented readers with strongly constraining (1a) and weakly constraining (1b) sentences in which the plausible target word was either the predictable completion of the strongly constraining context (“*church*”), unpredictable but semantically related to the best completion (“*sermon*”), or unpredictable but semantically unrelated to the best completion (“*garden*”).

(1a) *The priest wondered how he could get more people to come to the* ***church/sermon/garden*** *even though it was raining.*

(1b) *The widow thought that it was a lovely* ***church/sermon/garden*** *even though it was cold.*

As expected, predictable completions like “*church*” received stronger processing benefits under conditions of high constraint, i.e., higher skipping rates, shorter fixation durations, and fewer regressions than the average of the other conditions. But there was no evidence that either of the unpredictable completions (i.e., “*sermon*” and “*garden*”) presented in place of these more expected competitors disrupted readers’ eye movements in strongly relative to weakly constraining contexts. Instead, unpredictable completions that were semantically related to the best completion received shorter total reading times and fewer first-pass regressions in the strongly constraining contexts (i.e., “*sermon*” in 1a), suggesting that these items were easier to integrate due to their semantic overlap with the most predictable completion (see Federmeier et al., 2002; Federmeier & Kutas, 1999; Thornhill & Van Petten, 2012 for similar ERP findings). Similar findings were obtained in a recent eye-movement study by Wong et al. (2022) in which unpredictable completions were presented in three-sentence passages that varied in whether the source of constraint violation originated from the global or local context.

Luke and Christianson (2016) also found no evidence of prediction error costs when readers were presented with a corpus of naturalistic text passages for which cloze probability values had been calculated for each word. Instead, as the cloze probability of the best completion increased, unexpected content words were processed more efficiently as indexed by a higher rate of skipping and fewer refixations (see also Andrews et al., 2022). More recently, however, Cevoli et al. (2022) reported evidence of a prediction error cost when analysing Luke and Christianson’s eye-movement data using two predictability metrics derived from a language model: “surprisal,” which refers to the degree of surprise when a target word is reached as indexed by its negative log cloze probability, and “entropy,” which refers to the degree of uncertainty before a target word is reached as indexed by the extent to which a context is neutral or constraining (see also Lowder et al., 2018). Specifically, first fixation durations were longer when high surprisal or “unexpected” targets were presented in low entropy contexts where it was possible to make a lexical prediction about upcoming text, suggesting that readers’ eye movements were immediately disrupted by the mismatch between the predicted word and the input eventually encountered. This early prediction error cost, however, was resolved soon after—gaze and total fixation durations were reduced for unexpected targets that were semantically related to the best completion, indicating that integration processes facilitated their subsequent processing. Cevoli et al. concluded that readers did rely on lexical prediction during online processing although the immediate consequences of violating these expectations appeared to be short-lived. Thus, eye-movement studies to date provide mixed evidence of the processing costs that would be expected to occur if readers make lexical predictions that subsequently turn out to be incorrect.

ERP studies, on the contrary, have linked disconfirmed predictions to additional neural activity in the form of a late frontal positivity that emerges 500–1,000 ms post-stimulus onset (see Van Petten & Luka, 2012 for a review). Plausible unexpected completions consistently yield this neural waveform in strongly constraining contexts where a more expected competitor is available (DeLong et al., 2012; Federmeier et al., 2007), which has led researchers to hypothesise that it captures the processing consequences of suppressing the previously expected completion (Federmeier et al., 2007; Kutas, 1993; Ness & Meltzer-Asscher, 2018). Because the late frontal positivity has also been observed for plausible unexpected completions in weakly to moderately constraining contexts where no strong predictions can be made (Freunberger & Roehm, 2016; Thornhill & Van Petten, 2012; Zirnstein et al., 2018), this neural waveform has also been hypothesised to reflect the revision of the unfolding discourse representation based on the novel unexpected input (Brothers et al., 2015, 2020; DeLong, Troyer, & Kutas, 2014). More generally, this late frontal positivity has been distinguished from a late parietal positivity that arises for unexpected completions that are anomalous in the sentence context (DeLong, Quante, & Kutas, 2014; Kuperberg et al., 2020), providing further evidence that it reflects the consequences of prediction violation rather than general linguistic incongruity. In other words, anomalous unexpected completions do not elicit the late frontal positivity because the underlying processes of suppression and/or revision are not necessary for completions that cannot be integrated into an unfolding discourse representation. In contrast to eye-movement studies then, ERP studies provide more consistent evidence that readers do generate specific lexical predictions during online processing because they are sensitive to the costs of misprediction, although methodological factors such as the word-by-word presentation format and slower presentation rate used in ERP studies could have also encouraged different strategic processes to that of normal reading. Nonetheless, taken together, there are clear discrepancies across studies and methodologies in the apparent consequences of encountering unexpected input in place of a more predictable completion.

Recent ERP evidence suggests that effects of lexical prediction are observable not just during the immediate processing of critical words but also downstream from their initial presentation (Hubbard et al., 2019; Lai et al., 2021; Rommers & Federmeier, 2018a, 2018b). For example, when Rommers and Federmeier (2018b) presented sentences completed by predictable or unpredictable completions that appeared three sentences later, both types of repeated words elicited a repetition effect at the N400 component relative to a word that had not been previously seen. The size of this repetition effect, however, was smaller for previously expected completions, leading Rommers and Federmeier to speculate that predictable words were encoded less thoroughly during their initial presentation resulting in a more impoverished downstream representation.

More relevant to this research is a subsequent study by Rommers and Federmeier (2018a), which revealed that readers’ lexical predictions were observable downstream even if they did not materialise and were replaced by less expected input. Readers in this study were presented unpredictable targets like “*hot*” in weakly constraining sentences like (2a), or equally unpredictable targets like “*dirty*” in strongly constraining sentences like (2b), which replaced the more expected completion “*hot*.” Three sentences later, the critical target “*hot*” was presented in an unconstraining sentence such as “*The proofreader asked her to replace the word* ***hot***,” which assessed readers’ processing of a repeated word when following sentences like (2a) or a disconfirmed prediction when following sentences like (2b).

(2a) *He was surprised when he found out that it was* ***hot***.

(2b) *Be careful, because the top of the stove is very* ***dirty***.

At initial presentation, unpredictable targets in strongly constraining contexts yielded the expected late frontal positivity, reflecting readers’ sensitivity to the disconfirmed prediction. Further downstream, repeated words elicited the expected repetition effect at the N400 component relative to a word being presented for the first time. However, disconfirmed predictions, which were not presented but merely expected also elicited a similar attenuated N400. Although the size of this N400 reduction was smaller than the repetition effect, Rommers and Federmeier concluded that previously predictable words were still active in readers’ memory even though their occurrence in the sentence had been disconfirmed. Hubbard et al. (2019) provided converging evidence using a word recognition task that revealed higher rates of false alarms to “lures” that were predicted but never presented compared with items that had not been previously seen.

These ERP findings, therefore, provide further evidence that readers generate specific lexical predictions during online processing because these expectations can linger and facilitate downstream processing even if they do not eventuate and are replaced by less expected input. Importantly, because unexpected input also initially elicited the late frontal positivity, it implies that these expectations were likely suppressed, and the existing discourse representation was likely revised based on the input actually encountered. As such, the fact that previously predictable words were facilitated downstream suggests that the effects of misprediction were only temporary because these expectations subsequently lingered to affect processing. The idea that the processing consequences of misprediction are short-lived could account for why evidence of prediction error costs across previous eye-movement studies has been inconsistent, and generally elusive, during the immediate processing of unexpected input (Andrews et al., 2022; Cevoli et al., 2022; Frisson et al., 2017; Luke & Christianson, 2016; Wong et al., 2022). Accordingly, it raises the possibility that readers’ expectations could also remain active downstream in the eye-movement record even if they are disconfirmed by unexpected input.

Thus, the aim of the present set of experiments was two-fold. The first aim was to address inconsistencies in previous eye-movement investigations of anticipatory prediction by testing whether readers’ lexical predictions are observable during the immediate processing of critical words. The second aim was to extend these existing investigations by assessing evidence of anticipatory prediction that may be observable downstream from the initial presentation of critical words. The experiments presented short, connected sentence pairs in which the first sentence contained a target word that either confirmed or disconfirmed readers’ expectations, whereas the second sentence presented previously predictable words close to their initial point of activation. This design allowed us to assess prediction error costs at target words that have not been observed in previous eye-movement studies using controlled experimental designs (Frisson et al., 2017; Wong et al., 2022), and, more specifically, to determine whether readers’ predictions have downstream consequences even if they do not eventuate and are replaced by less expected input.

## Experiment 1

Readers’ eye movements were recorded as they read two-sentence passages. The first sentence was either strongly constraining towards a specific word or weakly constraining. The initial target word was either the predictable word or an unpredictable word (see Table 1 for an example item pair). Unpredictable words were either semantically related to the best completion of the strongly constraining context, semantically unrelated to the best completion, or syntactically and semantically anomalous within the sentence context. The relatedness manipulations allowed for investigation of whether the processing costs for unexpected input were modulated by the available semantic information (Frisson et al., 2017). The anomaly manipulation allowed for investigation of whether the suppression and/or revision processes that would be expected to accompany unexpected input were disrupted when the actual input presented did not fit within the overall discourse representation (Kutas, 1993; Ness & Meltzer-Asscher, 2018).

**Table 1.** Example set of items and mean (and standard deviation) stimulus characteristics.

Condition	Example item (Initial target bolded; Downstream target underlined)	Initial target cloze probability	Initial target frequency (logHAL)	Initial target length (letters)	Initial target sentence plausibility (1–5 scale)	Initial target relatedness to predictable word (LSA)	Constraint of first sentence
Strongly constraining context							
Predictable	Irene and her husband travelled by boat to the tropical **island** for their honeymoon. It was close to the island they had chosen for their wedding	.81 (.13)	9.5 (1.4)	5.4 (1.3)	4.9 (0.2)	1 (0)	.81 (.13)
Related	Irene and her husband travelled by boat to the tropical **resort** for their honeymoon. It was close to the island they had chosen for their wedding	.01 (.03)	8.8 (1.9)	5.3 (1.3)	4.6 (0.6)	0.3 (0.2)	.81 (.13)
Unrelated	Irene and her husband travelled by boat to the tropical **garden** for their honeymoon. It was close to the island they had chosen for their wedding.	.00 (.02)	9.4 (1.8)	5.4 (1.2)	4.2 (0.8)	0.1 (0.1)	.81 (.13)
Anomalous	Irene and her husband travelled by boat to the tropical **seeing** for their honeymoon. It was close to the island they had chosen for their wedding.	.00 (.00)	8.9 (2.2)	5.4 (1.3)	1.1 (0.2)	0.1 (0.1)	.81 (.13)
Weakly constraining context							
Predictable	Today we visited a beautiful **island** famous for exotic birds. Tomorrow, we will go to the island where the capital is located	.01 (.04)	9.5 (1.4)	5.4 (1.3)	4.7 (0.5)	1 (0)	.18 (.07)
Related	Today we visited a beautiful **resort** famous for exotic birds. Tomorrow, we will go to the island where the capital is located	.01 (.04)	8.8 (1.9)	5.3 (1.3)	4.6 (0.6)	0.3 (0.2)	.18 (.07)
Unrelated	Today we visited a beautiful **garden** famous for exotic birds. Tomorrow, we will go to the island where the capital is located	.01 (.05)	9.4 (1.8)	5.4 (1.2)	4.6 (0.6)	0.1 (0.1)	.18 (.07)
Anomalous	Today we visited a beautiful **seeing** famous for exotic birds. Tomorrow, we will go to the island where the capital is located	.00 (.00)	8.9 (2.2)	5.4 (1.3)	1.2 (0.3)	0.1 (0.1)	.18 (.07)

The anomalous conditions were not included in Experiments 2 or 3. The predictable conditions were not included in Experiment 3.

LSA: latent semantic analysis.

On the basis of previous eye-movement studies using similar controlled experimental designs (Frisson et al., 2017; Wong et al., 2022), predictable words in strongly constraining contexts were expected to elicit the largest processing benefits relative to the same words in weakly constraining contexts. However, no immediate consequences of prediction failure were expected when plausible unpredictable words were presented in strongly compared with weakly constraining contexts, regardless of their semantic relatedness to the best completion. Instead, related unpredictable words under conditions of strong constraint were expected to elicit processing benefits on late eye-movement measures due to their semantic overlap with the best completion. Anomalous words, on the contrary, were expected to elicit processing costs in both context conditions due to their overall linguistic incongruity (Braze et al., 2002; Rayner et al., 2004; Veldre & Andrews, 2016; Veldre et al., 2020) rather than to the violation of readers’ predictions per se.

Immediately following the first sentence, readers were presented with a thematically related unconstraining sentence that probed the downstream activation of the predictable word from the initial sentence as a function of whether it had been confirmed or disconfirmed. The downstream target word was therefore either a repeated word when readers previously encountered the predictable word in either of the context conditions, or a new word when readers previously encountered any of the unpredictable words, although this new word would have been previously expected in the strongly constraining contexts. These connected sentence pairs ensured that, unlike previous repetition paradigms used in ERP studies (Rommers & Federmeier, 2018a, 2018b), readers’ processing of the downstream target words relative to their initial point of activation was not delayed by several unrelated intervening sentences. However, the second sentence was always neutrally constraining to ensure that readers did not generate any other predictions that could interfere with the downstream activation of the previously predictable word.

Downstream targets that were repeated words were expected to yield different processing patterns following predictable completions in strongly compared with weakly constraining contexts. If predictable words are encoded less thoroughly because they simply confirm readers’ expectations (Rommers & Federmeier, 2018b), repeated words may yield repetition costs following predictable words in strongly compared with weakly constraining contexts. However, if predictable words are processed more thoroughly because their preactivation ahead of time frees up more of readers’ cognitive resources, repeated words may yield repetition benefits following predictable words under conditions of strong constraint.

Furthermore, if readers make predictions about upcoming words that involve the prediction of a specific lexical item (DeLong, Troyer, & Kutas, 2014), downstream targets that were new words were expected to yield different processing patterns following plausible unpredictable completions in strongly compared with weakly constraining contexts given that this word would have been previously expected, although never presented, in strongly constraining contexts. If the consequences of misprediction are short-lived, previously predictable words should still have active representations downstream despite being temporarily suppressed. As such, facilitated processing should be observed for new words following plausible unpredictable words in strongly but not weakly constraining contexts. But if the consequences of misprediction are long-lasting, previously predictable words should have suppressed representations downstream owing to more persistent inhibition processes (Kutas, 1993; Ness & Meltzer-Asscher, 2018). As such, inhibited processing should be observed for new words following plausible unpredictable words in strongly but not weakly constraining contexts.

If readers do not make predictions that involve a specific lexical item because they preactivate upcoming morphosyntactic, syntactic, and semantic information instead (Luke & Christianson, 2016), downstream targets that were new words were only expected to receive facilitated processing following unpredictable words that are semantically related to the best completion in strongly constraining contexts. Conversely, new words should be processed equivalently following unrelated unpredictable words in both context conditions given minimal semantic overlap with the best completion.

Finally, regardless of the type of predictions readers make about upcoming words, downstream targets that were new words were expected to receive facilitated processing following anomalous unpredictable words in strongly constraining contexts—the previously predictable word should not be suppressed given that anomalous words cannot be integrated into the unfolding discourse representation in the first place (Kutas, 1993).

### Methods

#### Participants

Sixty-two undergraduates from The University of Sydney participated in the eye-tracking task in return for course credit. The data from three participants were removed due to self-reported dyslexia, calibration difficulty, and comprehension accuracy in the eye-tracking task that was three standard deviations (*SD*s) below the mean. Therefore, the final sample comprised 59 participants (34 females; *M*_age_ = 20.2 years). All were native English speakers and had normal or corrected-to-normal vision. This research was approved by the University of Sydney Human Research Ethics Committee, and all participants provided written informed consent prior to participating in the study.

#### Materials

The critical stimuli were 76 pairs of two-sentence passages. Each pair was constructed such that in the strongly constraining passage, the initial target was high in predictability, whereas in the weakly constraining passage, the same initial target was low in predictability. Predictable words were compared with length- and frequency-matched unpredictable words that were either semantically related or unrelated to the predictable word, or anomalous in the sentence context. The second sentence was identical across all conditions and always contained the downstream target, i.e., the predictable initial target from the strongly constraining context.

The constraint of the first sentence and the predictability of the initial target was confirmed by cloze completions collected from a separate sample of 17 participants who did not complete the eye-tracking task (11 females; *M*_age_ = 20.1 years). Plausibility ratings of the first sentence were also collected to ensure that the related and unrelated unpredictable words were equivalently plausible continuations in both constraint conditions—a separate sample of 80 participants (47 females; *M*_age_ = 52.9 years) judged the plausibility of the entire first sentence on a five-point scale from 1 (*Highly Implausible*) to 5 (*Highly Plausible*). The semantic relatedness of the initial target was assessed by computing latent semantic analysis (LSA; Landauer & Dumais, 1997) scores between the predictable word and each of the unpredictable words. Table 1 presents an example item pair with the mean lexical characteristics of the target words for each condition.

#### Apparatus

Participants read the passages on a 21-inch ViewSonic G225f CRT monitor that was set to a pixel resolution of 1,024 × 768 and a 140 Hz refresh rate while their eye movements were tracked by an SR Research Eyelink 1000 eye-tracker, which had a sampling rate of 1,000 Hz. Passages were presented across two double-spaced lines in 14pt Consolas black font on a white background. Target words were never positioned at the beginning or end of a line. Participants were seated 60 cm from the monitor with a chin and forehead rest used to minimise head movements. At this distance, one degree of visual angle equated to 2.85 letter spaces. Viewing was binocular, but eye movements were recorded from participants’ right eye.

#### Procedure

Participants were instructed to read the passages for meaning and to respond to comprehension questions, which appeared after approximately 34% of the trials (mean accuracy = 90.5%).^1^ A nine-point calibration procedure was conducted before the start of the experiment. If mean calibration error was greater than 0.5° of visual angle, an additional calibration procedure was carried out. Before each trial, a fixation point appeared at the location of the first letter of the passage and a stable fixation on this point was required before the trial was displayed.

The passages were counterbalanced across four lists using a Latin square design so that each participant always saw a different target word in the strongly and weakly constraining version of each pair. Across all 152 passages, participants saw an equal number of target words per condition. Participants were randomly assigned to a list that randomly presented the passages across four equal blocks interspersed with 30 neutrally constraining filler passages.

The experimental materials, data, and analysis code for all experiments reported in this article are publicly available on the Open Science Framework website: https://osf.io/5rgck/.

### Results

Fixations shorter than 80 ms were automatically merged with adjacent fixations within one-letter space (0.35% of total fixations). Trials were removed if a participant prematurely ended the trial (0.2% of trials), or if there was track loss or blinks on the region of interest (Initial target: 5.3% of trials; Downstream target: 2.2% of trials). Fixations on the initial and downstream targets below 80 ms, first fixation durations above 800 ms, gaze durations above 1,200 ms, and total fixation durations above 2,000 ms were also excluded (Initial target: 1.3% of trials; Downstream target: 1.4% of trials). These exclusions left 8,357 initial target datapoints (93.2% of the data) and 8,619 downstream target datapoints (96.1% of the data) for analysis.

For both the initial and downstream targets, the following log-transformed reading measures were analysed: first fixation duration (the duration of the first fixation on a region), gaze duration (the sum of all fixations on a region before the eyes exit this region for the first time), and total fixation duration (the sum of all fixations on a region). The probability of skipping, regressions out of the target word to earlier in the text, and regressions into the target word from later in the text were also analysed. The average reading measures on the initial and downstream targets for each condition are presented in Table 2.

**Table 2.** Mean (and standard deviation) reading measures on the initial and downstream target words for each condition in Experiment 1.

	Reading measure	Strongly constraining context				Weakly constraining context			
		Predictable initial target	Related initial target	Unrelated initial target	Anomalous initial target	Predictable initial target	Related initial target	Unrelated initial target	Anomalous initial target
Target word	Skipping (%)	30 (12)	26 (10)	24 (11)	23 (11)	21 (9)	24 (9)	22 (9)	21 (11)
	First fixation (ms)	216 (26)	231 (25)	227 (22)	255 (28)	222 (22)	231 (19)	230 (22)	244 (25)
	Gaze (ms)	237 (38)	254 (28)	254 (28)	295 (40)	242 (32)	258 (27)	251 (28)	283 (35)
	Total fixation (ms)	279 (79)	316 (60)	343 (63)	594 (123)	316 (55)	354 (52)	349 (56)	598 (137)
	Regressions-out (%)	10 (7)	11 (9)	13 (7)	19 (11)	11 (7)	11 (8)	11 (6)	19 (11)
	Regressions-in (%)	7 (9)	14 (9)	17 (8)	52 (16)	19 (9)	25 (9)	25 (10)	58 (13)
Downstream word	Skipping (%)	35 (9)	36 (8)	34 (10)	35 (10)	33 (11)	32 (11)	32 (10)	28 (10)
	First fixation (ms)	204 (20)	205 (22)	208 (22)	208 (26)	206 (24)	203 (26)	214 (22)	207 (23)
	Gaze (ms)	226 (24)	224 (28)	227 (31)	229 (32)	233 (29)	230 (33)	241 (33)	236 (28)
	Total fixation (ms)	286 (41)	279 (34)	288 (37)	282 (40)	297 (42)	289 (45)	307 (45)	287 (40)
	Regressions-out (%)	15 (8)	15 (9)	16 (8)	16 (8)	16 (8)	19 (9)	18 (9)	19 (9)
	Regressions-in (%)	23 (7)	20 (8)	21 (8)	19 (9)	21 (9)	20 (9)	22 (10)	18 (9)

The data were analysed by (generalised) linear mixed effects models (GLMM/LMM) using the *lme4* package (Version 1.1-30; Bates et al., 2015) in *R*. The models tested the fixed effect of constraint nested under initial target type, which returned estimates of the constraint effect separately for predictable, related, unrelated, and anomalous words. The effect of constraint for the predictable words was equivalent to testing the benefit of making a correct prediction because these words were high cloze in the strongly constraining context but low cloze in the weakly constraining context. Meanwhile, the effect of constraint for each of the unpredictable words was equivalent to testing the cost of making an incorrect prediction because these words disconfirmed a more expected completion in the strongly constraining context but not in the weakly constraining context. The models also tested the main effect of initial target type, which was coded as a set of three orthogonal contrasts: (1) the predictability effect—the difference between the predictable condition and the two plausible unpredictable conditions, (2) the relatedness effect—the difference between the related unpredictable condition and the unrelated unpredictable condition, and (3) the anomaly effect—the difference between the anomalous condition and the three plausible conditions. Given that these contrasts are averaged over constraint, they are not directly relevant to the interpretation of the initial target; but their inclusion is important for the purpose of accounting for variance in the models (Schad et al., 2020). These contrasts, however, are relevant to the interpretation of the downstream target because the predictability effect is equivalent to testing the repeated word effect (i.e., the difference between the repeated and new words), whereas the relatedness and anomaly effects are equivalent to testing the new word effect (i.e., the difference between the new words depending on the initial target presented). Thus, the outcomes of these contrasts are reported for both target words, but interpretations are restricted to the downstream target.

All models either failed to converge or showed singular fit with the maximal random-effects structure (i.e., subject and item random intercepts and random slopes for the nested constraint effect under each level of initial target type). Therefore, the random-effects structure for each model was simplified: first by removing the correlation parameters between random intercepts and random slopes, and second by sequentially removing random slopes that accounted for the least variance until model convergence without singular fit. Estimates yielding *t*/*z* values greater than|1.96| were interpreted as significant at the .05 α level. Power analyses conducted with 100 Monte Carlo simulations using the *simR* package (Version 1.0.6; Green & MacLeod, 2016) in *R* demonstrated adequate power to detect the constraint effect for predictable initial targets (> .97) and related initial targets (> .94) based on comparable effects reported in the study by Frisson et al. (2017; predictable targets: 16 ms on first fixation duration, 24 ms on gaze duration; 63 ms on total fixation duration; related targets: 51 ms on total fixation duration). The models also had adequate power (> .80) to detect the constraint effect for downstream targets following each of the unpredictable words of 11 ms effect size on first fixation duration, 15 ms effect size on gaze duration, and 25 ms effect size on total fixation duration. Summaries of the statistical analyses for the initial and downstream targets are presented in Table 3.^2^

**Table 3.** Results for the nested linear mixed effects models for log-transformed fixation duration measures and generalised linear mixed effects models for fixation probability measures on the initial and downstream target words in Experiment 1.

Measure	Fixed effect	Initial target word			Downstream target word		
		*b*	*SE*	*t*/*z*	*b*	*SE*	*t*/*z*
Skipping	Intercept	***–*1.30**	**0.10**	***–*12.60**	***–*0.78**	**0.10**	***–*7.80**
	Predictability	0.11	0.07	1.63	0.04	0.06	0.59
	Relatedness	0.10	0.08	1.25	0.05	0.07	0.72
	Anomaly	***–*0.14**	**0.06**	***–*2.20**	*–*0.11	0.06	*–*1.90
	Predictable target: Constraint effect	***–*0.52**	**0.13**	***–*4.11**	*–*0.09	0.15	*–*0.62
	Related target: Constraint effect	*–*0.14	0.13	*–*1.08	*–*0.22	0.11	*–*1.93
	Unrelated target: Constraint effect	*–*0.15	0.11	*–*1.35	*–*0.13	0.14	*–*0.93
	Anomalous target: Constraint effect	*–*0.13	0.12	*–*1.12	***–*0.37**	**0.14**	***–*2.60**
First fixation	Intercept	**5.39**	**0.02**	**314.78**	**5.27**	**0.02**	**298.98**
	Predictability	***–*0.05**	**0.01**	***–*5.07**	*–*0.01	0.01	*–*1.02
	Relatedness	0.01	0.01	0.66	***–*0.03**	**0.01**	***–*2.36**
	Anomaly	**0.10**	**0.01**	**11.02**	0.00	0.01	0.41
	Predictable target: Constraint effect	**0.03**	**0.02**	**1.99**	*–*0.01	0.03	*–*0.32
	Related target: Constraint effect	*–*0.00	0.02	*–*0.25	*–*0.01	0.02	*–*0.54
	Unrelated target: Constraint effect	0.02	0.02	0.76	0.01	0.02	0.27
	Anomalous target: Constraint effect	***–*0.04**	**0.02**	***–*2.11**	*–*0.01	0.02	*–*0.38
Gaze	Intercept	**5.47**	**0.02**	**276.26**	**5.36**	**0.02**	**279.11**
	Predictability	***–*0.06**	**0.01**	***–*5.37**	*–*0.01	0.01	*–*0.43
	Relatedness	0.01	0.01	0.68	*–*0.02	0.01	*–*1.53
	Anomaly	**0.13**	**0.01**	**12.56**	0.01	0.01	0.68
	Predictable target: Constraint effect	0.03	0.02	1.31	0.00	0.03	0.07
	Related target: Constraint effect	0.00	0.02	0.19	0.02	0.03	0.62
	Unrelated target: Constraint effect	*–*0.01	0.02	*–*0.34	0.04	0.03	1.26
	Anomalous target: Constraint effect	*–*0.05	0.03	*–*1.76	0.03	0.03	0.88
Total fixation	Intercept	**5.78**	**0.03**	**200.20**	**5.53**	**0.02**	**225.39**
	Predictability	***–*0.13**	**0.01**	***–*8.87**	0.00	0.01	0.27
	Relatedness	*–*0.02	0.02	*–*1.32	***–*0.04**	**0.02**	***–*2.33**
	Anomaly	**0.57**	**0.01**	**43.86**	*–*0.01	0.01	*–*0.51
	Predictable target: Constraint effect	**0.12**	**0.03**	**4.14**	0.01	0.04	0.28
	Related target: Constraint effect	**0.10**	**0.03**	**3.14**	0.03	0.03	0.98
	Unrelated target: Constraint effect	0.03	0.03	0.79	0.06	0.03	1.92
	Anomalous target: Constraint effect	0.02	0.03	0.56	0.01	0.03	0.41
Regressions-out	Intercept	***–*2.06**	**0.09**	***–*22.41**	***–*1.78**	**0.09**	***–*19.98**
	Predictability	*–*0.09	0.09	*–*1.01	*–*0.03	0.08	*–*0.33
	Relatedness	*–*0.08	0.10	*–*0.85	*–*0.00	0.09	*–*0.02
	Anomaly	**0.67**	**0.07**	**9.45**	0.07	0.07	1.01
	Predictable target: Constraint effect	0.15	0.15	1.01	0.13	0.17	0.76
	Related target: Constraint effect	0.07	0.20	0.37	0.26	0.20	1.28
	Unrelated target: Constraint effect	*–*0.20	0.14	*–*1.42	0.22	0.20	1.08
	Anomalous target: Constraint effect	*–*0.07	0.15	*–*0.48	0.27	0.19	1.46
Regressions-in	Intercept	***–*1.25**	**0.10**	***–*12.76**	***–*1.70**	**0.13**	***–*12.74**
	Predictability	***–*0.67**	**0.08**	***–*7.92**	0.12	0.07	1.65
	Relatedness	*–*0.14	0.08	*–*1.69	*–*0.13	0.09	*–*1.47
	Anomaly	**1.98**	**0.06**	**32.54**	***–*0.23**	**0.07**	***–*3.01**
	Predictable target: Constraint effect	**1.13**	**0.17**	**6.57**	*–*0.14	0.20	*–*0.68
	Related target: Constraint effect	**0.81**	**0.14**	**5.97**	*–*0.04	0.25	*–*0.16
	Unrelated target: Constraint effect	**0.47**	**0.14**	**3.27**	0.04	0.22	0.19
	Anomalous target: Constraint effect	**0.27**	**0.12**	**2.24**	*–*0.08	0.25	*–*0.34

Significant effects are bolded.

#### Initial target

The predictability effect was significant on all reading measures except skipping (*z* = 1.63) and regressions-out (*z* = –1.01) because, averaged over constraint, predictable targets received shorter fixation durations (|*t*|s > 5.07) and fewer regressions-in (*z* = –7.92) compared with plausible unpredictable targets. Although the relatedness effect was not significant on any reading measures (|*t*/*z*|s < 1.69), the anomaly effect was significant on all reading measures (|*t*/*z*|s > 2.20) because, averaged over constraint, readers showed lower skipping rates, longer fixation durations, and more regressions for anomalous relative to plausible targets.

For predictable targets, the constraint effect was significant on skipping, first fixation and total fixation duration, and regressions-in (|*t*/*z*|s > 1.99) because predictable targets received higher skipping rates, shorter reading times, and fewer regressions-in when presented in strongly compared with weakly constraining contexts. For related unpredictable targets, the constraint effect was significant on total fixation duration and regressions-in (|*t*/*z*|s > 3.14) reflecting shorter total reading times, and fewer regressions-in for related unpredictable targets under conditions of strong compared with weak constraint. For unrelated unpredictable targets, the facilitatory constraint effect was restricted to regressions-in (*z* = 3.27) due to fewer regressions-in from later parts of the text in strongly compared with weakly constraining contexts. Finally, for anomalous unpredictable targets, the constraint effect was significant on first fixation duration (*t* = –2.11), and regressions-in (*z* = 2.24) due to longer initial fixations but fewer regressions-in for anomalous unpredictable targets under conditions of strong compared with weak constraint.

Thus, as expected, predictable targets showed the largest predictability benefits in strongly constraining contexts. Plausible unpredictable targets that disconfirmed these expectations also received facilitated, rather than slowed, processing on late reading measures in strongly compared with weakly constraining contexts. The only evidence of predictability costs was restricted to anomalous completions presented under conditions of strong constraint.

#### Downstream target

The predictability effect was not significant on any of the reading measures at the downstream target (i.e., the predictable word from the initial sentence;|*t*/*z*|s < 1.65) because repeated words following predictable words were processed equivalently to new words following any of the plausible unpredictable words. The relatedness effect was significant on first fixation and total fixation duration (|*t*|s > 2.33) because new words received shorter fixation durations when following related compared with unrelated words. The anomaly effect was significant on regressions-in (*z* = –3.01) because new words received fewer regressions-in when following anomalous compared with plausible words. Thus, downstream repeated targets were processed equivalently to downstream new targets, although the latter showed some processing facilitation depending on the completion that appeared in the first sentence.

For downstream targets following predictable words, the constraint effect was not significant on any reading measures (|*t*/*z*|s < 1) because repeated words were processed equivalently following predictable words in the initial sentence across both constraint conditions. For downstream targets following related or unrelated words, the constraint effect was not significant on any reading measures (|*t*/*z*|s < 1.93) as new words were processed equivalently following related or unrelated words in the initial sentence across both constraint conditions. Finally, for downstream targets following anomalous words, the constraint effect was significant on skipping (*z* = –2.60) because new words were more likely to be skipped if the anomalous word in the initial sentence was presented under conditions of strong compared with weak constraint.

Thus, downstream repeated targets were processed equivalently when following a predictable completion in strongly compared with weakly constraining contexts. Similarly, downstream new targets were not processed differently when following plausible but unpredictable completions under conditions of strong constraint. However, downstream new targets were more likely to be skipped when following an anomalous unpredictable completion in strongly constraining contexts.

### Discussion

The aim of this experiment was to go beyond existing eye-movement investigations of prediction error costs (Frisson et al., 2017; Wong et al., 2022) by examining whether lexical prediction has observable immediate and/or downstream consequences even if readers’ expectations do not materialise and are replaced by less expected input. Consistent with previous eye-movement findings (Andrews et al., 2022; Frisson et al., 2017; Luke & Christianson, 2016; Wong et al., 2022), at initial presentation, there was no indication that plausible unexpected input in strongly constraining contexts elicited immediate processing costs despite violating a more expected completion. The only evidence that disconfirmed predictions disrupted readers’ eye movements was when unexpected input was also syntactically and semantically incongruous in the sentence context. Specifically, anomalous unpredictable targets in strongly compared with weakly constraining contexts elicited longer first fixation durations, although the rate of regressions-in from later in the text was subsequently reduced, reflecting short-lived processing costs that were resolved across later eye-movement measures. However, the fact that this processing disruption was limited to anomalous input suggests that, rather than being due to the violation of readers’ predictions, it was more likely due to the linguistic incongruity between the input and the context, which was detected more rapidly under conditions of strong constraint.

Following this initial sentence, a thematically related unconstraining sentence containing the predictable completion from the initial sentence was presented to probe the downstream activation of previously confirmed and disconfirmed predictions. Surprisingly, downstream targets that were repeated words did not appear to be processed more efficiently than downstream targets that were new words, suggesting that readers did not benefit from encountering the same input more than once. Moreover, contrary to our hypotheses, downstream repeated words did not appear to be processed differently following the most expected completion in strongly compared with weakly constraining contexts, leaving open the question of how thoroughly predictable words in the first sentence were processed, i.e., whether predictable words were processed less thoroughly because they simply confirmed readers’ expectations (Rommers & Federmeier, 2018b) or more thoroughly because their preactivation ahead of time allowed readers to devote more cognitive resources.

New downstream targets, on the contrary, yielded processing benefits on first fixation and total fixation duration when following a related compared with unrelated unpredictable completion, suggesting that readers benefitted from encountering input that shared semantic overlap with an earlier completion. New downstream targets also received fewer regressions-in when following an anomalous compared with plausible completion. But the only evidence that downstream targets presented for the first time were affected by the presence of a previously predictable, but never presented, completion was on skipping rates—new downstream targets were more likely to be skipped following anomalous completions in strongly constraining contexts where readers were encouraged to make a specific lexical prediction compared with weakly constraining contexts where they were not. These facilitatory effects on new downstream targets following anomalous unpredictable completions were expected because these items, which could not be integrated, were unlikely to interfere with the incorrectly predicted word (Kutas, 1993). However, the lack of any downstream effects on new downstream targets following either the related or unrelated unpredictable completions under conditions of strong constraint suggests that readers may not have actually generated any predictions about the upcoming text, reducing their sensitivity to encountering what was previously an incorrect prediction.

The finding that reading times at the downstream word showed no effects of repetition but did show effects of relatedness is somewhat surprising. Repetition effects, i.e., decreased processing times for a word presented more than once, have been consistently observed across a number of eye-movement studies of natural reading (Hyönä & Niemi, 1990; Kamienkowski et al., 2018; Raney & Rayner, 1995), and are often accompanied by increases in saccade length and decreases in the number of regressive saccades. Repetition effects have also been reported in behavioural tasks such as lexical decision (Scarborough et al., 1977) and naming (Lowder et al., 2013; Masson & Freedman, 1990), although these tasks clearly differ from normal reading in many aspects. In contrast, semantic priming effects, i.e., processing facilitation for a word preceded by a semantically related word, remain elusive in eye-movement studies. Furthermore, these effects have been shown to be constrained by syntactic information in the sentence such as clausal boundaries and linguistic focus (Carroll & Slowiaczek, 1986; Morris & Folk, 1998) or easily overriden by message-level information in the sentence such as plausibility and the presence of discourse context (Camblin et al., 2007; Traxler et al., 2000). Semantic priming effects, however, have been reported in behavioural tasks that involve the recognition of words in isolation such as lexical decision (Meyer & Schvaneveldt, 1971; Neely, 1976) and naming (Hutchison et al., 2013). Thus, one potential explanation for the pattern of effects observed in the current experiment is that, due to some aspect of the task or linguistic environment, i.e., the presence of anomalous completions, readers altered their normal reading strategies and processed the two-sentence passages as two separate, unconnected texts despite their thematic relationship, leading to the presence of semantic priming effects but the absence of repetition effects.

More generally, the absence of robust downstream consequences following either confirmed or disconfirmed predictions could be explained by the weaker than expected predictability effects observed at the target word in the initial sentence. Previous eye-movement studies have shown that cloze probability reliably influences readers’ earliest fixations on a word as indexed by first fixation and gaze duration (Balota et al., 1985; Fitzsimmons & Drieghe, 2013; Rayner & Well, 1996; but see Calvo & Meseguer, 2002; Hyönä, 1993), and sometimes even before readers make these fixations as indexed by skipping rates (Balota et al., 1985; Rayner et al., 2011; Rayner & Well, 1996) and parafoveal processing of upcoming words (Schotter et al., 2015; Veldre & Andrews, 2018). In this experiment, predictable targets in strongly constraining contexts received facilitated processing across several reading measures, but, on early measures, this effect was only significant on skipping, and just reached significance on first fixation duration because of a relatively small benefit (6 ms effect). The absence of strong predictability effects is unlikely to be due to a weak manipulation of target predictability because the average cloze probability of the predictable completions in the strongly constraining contexts was very high (.81). Meanwhile, the same completions in the weakly constraining contexts were very rarely produced (.02).

Instead, the failure to observe robust first-pass predictability effects in the strongly constraining contexts could be because the presence of linguistic incongruity disrupted readers’ normal processing strategies. Previous research has shown that severe violations of plausibility yield immediate processing difficulties during reading (e.g., “He used a pump to inflate the large carrots for dinner”; Rayner et al., 2004; see also Braze et al., 2002; Veldre & Andrews, 2016; Veldre et al., 2020). As such, the inclusion of anomalous targets in the present experiment, which comprised 25% of the critical stimuli, may have implicitly discouraged readers from generating strong lexical predictions about upcoming words, resulting in the weaker than expected context-specific predictability effects on reading measures at the target and downstream words. Accordingly, Experiment 2 investigated the same research questions as Experiment 1, but removed the anomalous condition to create a more naturalistic linguistic environment for readers.

## Experiment 2

Experiment 2 was designed to investigate the immediate and downstream consequences of lexical prediction in the eye-movement record without the inclusion of anomalous completions that may have limited the extent to which readers actively committed to a specific lexical prediction in Experiment 1. If readers are encouraged to rely more strongly on lexical prediction during online processing, this should be reflected in stronger effects of constraint for predictable targets especially on first-pass reading measures, and subsequently in downstream effects for confirmed and disconfirmed predictions.

### Methods

#### Participants

Sixty-five undergraduates from The University of Sydney who did not complete any part of Experiment 1 took part in the eye-tracking task in return for course credit. The data of five participants were removed due to calibration difficulty in the eye-tracking task. Therefore, the final sample comprised 60 participants (42 females; *M*_age_ = 22.3 years). All were native English speakers and had normal or corrected-to-normal vision.

#### Materials

The critical stimuli were the same 76 pairs of two-sentence passages used in Experiment 1. Each pair comprised a strongly constraining and weakly constraining passage, which was completed either by the predictable word for the strongly constraining context, or by a semantically related or unrelated alternative. The second sentence was identical across all conditions and contained the downstream target, which was the predictable initial target from the strongly constraining context. The lexical characteristics of the target words for each condition were identical to Experiment 1.

#### Apparatus

There were no changes in the apparatus from Experiment 1.

#### Procedure

The procedure was identical to Experiment 1 except that the 152 passages were counterbalanced across three lists. Comprehension questions appeared after approximately 34% of the trials (mean accuracy = 94.4%).

### Results

Data handling was the same as Experiment 1. Fixations shorter than 80 ms were automatically merged with adjacent fixations within one-letter space (0.26% of total fixations). Trials were removed if there was track loss or blinks on the region of interest (Initial target: 5.9% of trials; Downstream target: 2.7% of trials). Fixations on the initial and downstream targets below 80 ms, first fixation durations above 800 ms, gaze durations above 1,200 ms, and total durations above 2,000 ms were also excluded (Initial target: 0.7% of trials; Downstream target: 1.8% of trials). These exclusions left 8,515 initial target datapoints (93.4% of the data) and 8,711 downstream target datapoints (95.5% of the data) for analysis. The average reading measures on the initial and downstream targets for each condition are presented in Table 4.

**Table 4.** Mean (and standard deviation) reading measures on the initial and downstream target words for each condition in Experiment 2.

	Reading measure	Strongly constraining context			Weakly constraining context		
		Predictable initial target	Related initial target	Unrelated initial target	Predictable initial target	Related initial target	Unrelated initial target
Target word	Skipping (%)	33 (9)	28 (10)	28 (8)	27 (9)	25 (8)	27 (9)
	First fixation (ms)	207 (17)	221 (19)	221 (18)	210 (17)	221 (16)	213 (15)
	Gaze (ms)	223 (25)	245 (26)	243 (27)	230 (24)	247 (23)	234 (25)
	Total fixation (ms)	304 (51)	357 (58)	375 (47)	362 (51)	407 (63)	402 (59)
	Regressions-out (%)	11 (5)	15 (7)	14 (7)	16 (8)	16 (8)	15 (9)
	Regressions-in (%)	16 (9)	21 (7)	27 (10)	30 (10)	35 (10)	35 (10)
Downstream word	Skipping (%)	35 (10)	36 (9)	35 (8)	36 (9)	32 (9)	33 (10)
	First fixation (ms)	193 (18)	197 (18)	200 (21)	195 (16)	196 (17)	200 (19)
	Gaze (ms)	211 (22)	214 (20)	218 (25)	222 (24)	223 (25)	227 (27)
	Total fixation (ms)	300 (39)	299 (40)	311 (34)	323 (37)	315 (37)	326 (37)
	Regressions-out (%)	20 (7)	18 (7)	19 (8)	17 (7)	21 (8)	20 (7)
	Regressions-in (%)	26 (7)	28 (8)	29 (8)	29 (10)	27 (9)	29 (8)

As in Experiment 1, (G)LMMs were used to test the fixed effect of constraint nested under initial target type, which returned estimates of the main effect of initial target type and the constraint effect separately for predictable, related, and unrelated words. Initial target type was coded as a set of two orthogonal contrasts that tested the same predictability and relatedness effects as Experiment 1. As in the previous experiment, the outcomes of these contrasts are reported for both target words, but interpretations are restricted to the downstream target. Power analyses using the same procedure as Experiment 1 demonstrated adequate power to detect the constraint effect for predictable initial targets across the three fixation duration measures (> .99) and for related initial targets on total fixation duration (> .99) based on comparable effects reported in the study by Frisson et al. (2017). The models also had adequate power (> .80) to detect the constraint effect for downstream targets following each of the unpredictable words of 9 ms effect size on first fixation duration, 12 ms effect size on gaze duration, and 24 ms effect size on total fixation duration. Criteria for the random-effects structures and significance thresholds were identical to Experiment 1. Summaries of the statistical analyses for the initial and downstream targets are presented in Table 5.

**Table 5.** Results for the nested linear mixed effects models for log-transformed fixation duration measures and generalised linear mixed effects models for fixation probability measures on the initial and downstream target words in Experiment 2.

Measure	Fixed effect	Initial target word			Downstream target word		
		*b*	*SE*	*t*/*z*	*b*	*SE*	*t*/*z*
Skipping	Intercept	***–*1.05**	**0.10**	***–*10.21**	***–*0.76**	**0.12**	***–*6.22**
	Predictability	**0.16**	**0.05**	**3.02**	0.08	0.05	1.60
	Relatedness	*–*0.06	0.06	*–*1.02	0.00	0.06	0.03
	Predictable target: Constraint effect	***–*0.30**	**0.09**	***–*3.19**	0.02	0.12	0.15
	Related target: Constraint effect	*–*0.15	0.10	*–*1.44	*–*0.20	0.12	*–*1.67
	Unrelated target: Constraint effect	*–*0.05	0.10	*–*0.53	*–*0.08	0.12	*–*0.69
First fixation	Intercept	**5.32**	**0.02**	**317.62**	**5.21**	**0.02**	**279.76**
	Predictability	***–*0.05**	**0.01**	***–*6.57**	***–*0.02**	**0.01**	***–*2.45**
	Relatedness	0.02	0.01	1.79	***–*0.02**	**0.01**	***–*2.28**
	Predictable target: Constraint effect	0.02	0.02	1.26	0.00	0.02	0.16
	Related target: Constraint effect	0.00	0.01	0.30	*–*0.00	0.02	*–*0.16
	Unrelated target: Constraint effect	*–*0.03	0.02	*–*1.87	*–*0.01	0.02	*–*0.54
Gaze	Intercept	**5.39**	**0.02**	**273.62**	**5.31**	**0.02**	**251.26**
	Predictability	***–*0.07**	**0.01**	***–*7.09**	***–*0.02**	**0.01**	***–*1.98**
	Relatedness	**0.03**	**0.01**	**2.79**	***–*0.03**	**0.01**	***–*2.77**
	Predictable target: Constraint effect	0.03	0.02	1.73	0.04	0.03	1.18
	Related target: Constraint effect	0.01	0.02	0.30	0.02	0.03	0.79
	Unrelated target: Constraint effect	***–*0.04**	**0.02**	***–*2.07**	0.02	0.03	0.74
Total fixation	Intercept	**5.73**	**0.03**	**170.55**	**5.58**	**0.03**	**186.70**
	Predictability	***–*0.14**	**0.01**	***–*11.05**	*–*0.01	0.01	*–*1.16
	Relatedness	*–*0.01	0.01	*–*0.38	***–*0.04**	**0.01**	***–*3.14**
	Predictable target: Constraint effect	**0.17**	**0.03**	**5.43**	0.06	0.03	1.76
	Related target: Constraint effect	**0.11**	**0.03**	**3.56**	0.03	0.03	1.18
	Unrelated target: Constraint effect	**0.06**	**0.03**	**2.00**	0.03	0.03	1.07
Regressions-out	Intercept	***–*1.98**	**0.11**	***–*18.36**	***–*1.60**	**0.09**	***–*17.40**
	Predictability	*–*0.11	0.07	*–*1.55	*–*0.05	0.06	*–*0.88
	Relatedness	0.07	0.08	0.90	*–*0.03	0.07	*–*0.36
	Predictable target: Constraint effect	**0.56**	**0.13**	**4.20**	*–*0.19	0.16	*–*1.14
	Related target: Constraint effect	0.10	0.14	0.70	0.21	0.18	1.17
	Unrelated target: Constraint effect	0.12	0.17	0.70	0.07	0.16	0.46
Regressions-in	Intercept	***–*1.11**	**0.10**	***–*11.18**	***–*1.19**	**0.13**	***–*9.27**
	Predictability	***–*0.40**	**0.06**	***–*6.91**	*–*0.07	0.06	*–*1.24
	Relatedness	***–*0.17**	**0.06**	***–*2.80**	***–*0.14**	**0.07**	***–*2.14**
	Predictable target: Constraint effect	**0.91**	**0.13**	**6.73**	0.17	0.20	0.82
	Related target: Constraint effect	**0.75**	**0.13**	**5.84**	*–*0.10	0.19	*–*0.51
	Unrelated target: Constraint effect	**0.41**	**0.13**	**3.12**	*–*0.09	0.18	*–*0.49

Significant effects are bolded.

#### Initial target

The predictability effect was significant on all reading measures except regressions-out (*z* = –1.55) because, averaged over constraint, predictable targets received higher rates of skipping (*z* = 3.02), shorter fixation durations (|*t*|s > 6.57), and fewer regressions-in from later parts of the text (*z* = –6.91) than plausible unpredictable targets. The relatedness effect was significant on gaze duration and regressions-in (|*t*/*z*|s > 2.79) because, averaged over constraint, related unpredictable targets received longer gaze durations but fewer regressions-in than unrelated unpredictable targets.

For predictable targets, the constraint effect was significant on all reading measures except first fixation and gaze duration (|*t*|s < 1.73) because predictable targets received higher skipping rates (*z* = –3.19), shorter total reading times (*t* = 5.43), and fewer regressions-out and -in (|*z*|s > 4.20) when presented in strongly compared with weakly constraining contexts. For related unpredictable targets, there was a significant facilitatory constraint effect on total fixation duration and regressions-in (|*t*/*z*|s > 3.56) due to shorter total reading times, and fewer regressions-in for related unpredictable targets under conditions of strong compared with weak constraint. Finally, for unrelated unpredictable targets, the constraint effect was significant on gaze and total fixation duration (|*t*|s > 2.00) and regressions-in (*z* = 3.12) because unrelated unpredictable targets received longer first-pass reading times but subsequently shorter total reading times and fewer regressions-in under conditions of strong compared with weak constraint.

Thus, the current results were virtually identical to Experiment 1 with respect to predictable and related unpredictable targets, which were processed more efficiently in strongly constraining contexts. However, Experiment 2 provided novel evidence of a prediction error cost for first-pass reading of unrelated unpredictable targets under conditions of strong constraint.

#### Downstream target

The predictability effect was significant on first fixation and gaze duration at the downstream target (|*t*|s > 1.98) because repeated words received shorter fixation durations compared with new words. The relatedness effect was significant on all fixation duration measures (|*t*|s > 2.28) and regressions-in (*z* = –2.14) because new words received shorter fixation durations and fewer regressions-in when following related versus unrelated unpredictable words. The constraint effect was not significant on any of the reading measures at the downstream target regardless of the completion that appeared in the first sentence (|*t*/*z*|s < 1.76).

Thus, in contrast to Experiment 1, downstream repeated targets were processed more efficiently than downstream new targets, whereas the latter were also processed more efficiently when following a related than unrelated unpredictable completion in the initial sentence. But there was no evidence that downstream targets showed any processing consequences when following any of the initial targets in the strongly compared with weakly constraining contexts.

### Discussion

This experiment aimed to determine whether the weaker than expected immediate and downstream consequences of lexical prediction observed in Experiment 1 were due to the inclusion of anomalous completions that may have limited the extent to which readers actively committed to a specific lexical prediction. Experiment 2, therefore, removed anomalous completions to encourage a more naturalistic linguistic environment for readers.

The results revealed that, at initial presentation, predictable targets in strongly constraining contexts yielded the expected processing benefits in the form of higher skipping rates, shorter total reading times, and fewer regressions-out and -in compared to the same targets in weakly constraining contexts. Similar to Experiment 1, these facilitatory effects did not consistently affect early reading measures (i.e., first fixation and gaze duration), suggesting that the removal of anomalous completions did not enhance the immediate processing benefits for expected input. Nonetheless, these processing benefits did extend to semantically related alternatives—related unpredictable targets in strongly constraining contexts received shorter total reading times and fewer regressions-in, providing further evidence that unexpected input were easier to integrate when semantically compatible with the best completion.

However, contrary to the findings of Experiment 1, unpredictable targets that were semantically unrelated to the best completion elicited an immediate processing cost on gaze duration in strongly compared with weakly constraining contexts. This prediction error cost, which suggests that readers were immediately sensitive to the mismatch between the expected word and the input actually presented, was only temporary because unrelated unpredictable targets subsequently received shorter total reading times and fewer regressions-in from later parts of the text under conditions of strong constraint, implying that readers were able to resolve their incorrect predictions via integration processes that were supported by information extracted from the rest of the sentence and/or passage. Thus, these findings indicate that although evidence of immediate processing benefits for predictable targets was restricted to higher skipping rates only, readers did appear to make an active commitment to a specific lexical item because violation of these predictions elicited an immediate, albeit short-lived, processing cost.

This evidence of prediction error cost may have been obscured in Experiment 1 because the presence of anomalous completions discouraged readers from making strong lexical predictions. Supplementary analyses confirmed that, compared with Experiment 2, the magnitude of the predictability benefit was attenuated in Experiment 1 on the probability of regressions-out, as well as the magnitude of the predictability cost in Experiment 1 on first fixation duration.^3^ Compared with target words in Experiment 2, target words in Experiment 1 also received overall shorter total reading times, and fewer regressions-out and -in, suggesting that readers did not engage in late integration processes as thoroughly in the presence of linguistic incongruity.^4^ Thus, readers’ overall processing strategies, including those related to anticipatory prediction, appear to have differed between the two experiments.

Further downstream, the impact of removing anomalous completions was also evident. Consistent with previous eye-movement findings (Hyönä & Niemi, 1990; Lowder et al., 2013; Raney & Rayner, 1995), downstream targets that were repeated words because a predictable completion was previously encountered elicited the expected repetition benefit in the form of shorter first fixation and gaze durations compared with downstream targets that were new words because any of the unpredictable completions were previously encountered. However, like Experiment 1, there was no evidence that these repeated downstream targets were processed differently following a predictable completion in strongly compared with weakly constraining contexts, even though preactivation of the expected completion would have been greater under conditions of strong constraint. These findings suggest that the removal of anomalies may have contributed to a linguistic environment that encouraged readers to process the two-sentence passages as connected texts like they would during normal reading for comprehension, leading to enhanced sensitivity for repeated words. However, prior predictability did not appear to affect subsequent processing of these completions any further.

Moreover, similar to Experiment 1, downstream targets that were being encountered for the first time elicited processing benefits in the form of shorter fixation durations and fewer regressions-in when following a related versus unrelated unpredictable completion. But there was no evidence that these new downstream targets following either of the unpredictable completions were affected by the presence of a previously predictable, but never presented, word. This suggests that the processing benefits for new downstream targets following related completions most likely reflected their general semantic overlap. Thus, even under more naturalistic linguistic environments that elicited immediate processing costs for unexpected input, there appears to be no evidence that readers’ lexical predictions were observable downstream from their initial point of activation.

Therefore, the major novel finding of Experiment 2 was evidence of an immediate processing cost for unexpected input when semantically unrelated to the most expected competitor. Given that this prediction error cost did not emerge in Experiment 1, which presented the same sentence frames excluding the anomalous completions, it appears that the presence of linguistic incongruity may have implicitly discouraged readers from generating strong lexical predictions about upcoming words in the previous experiment. Nonetheless, the processing cost observed in this experiment was relatively small (9 ms effect) and restricted to a single eye-movement measure, which raises questions about its reliability (von der Malsburg & Angele, 2017). Accordingly, Experiment 3 was conducted to increase statistical power to detect a small effect by removing the predictable condition, thereby increasing the number of items in the critical unpredictable conditions.

## Experiment 3

Experiment 3 was designed to replicate and confirm the novel evidence of prediction error cost in Experiment 2 by increasing statistical power to detect this effect. Readers’ eye movements were recorded as they read strongly and weakly constraining sentences that contained plausible unpredictable words that were either semantically related or unrelated to the most expected completion of the strongly constraining context.

Following this sentence, readers were presented with a thematically related unconstraining sentence that contained either the predictable, but never presented, completion from the strongly constraining sentence or the other plausible, unpredictable completion. Readers’ eye-movement patterns on the downstream target are not reported below because, similar to the previous experiments, there were minimal downstream consequences following any of the completions that appeared in the first sentence (see Supplemental Materials for more details).

### Methods

#### Participants

Fifty-eight participants from The University of Sydney who did not complete any part of the previous experiments took part in the eye-tracking task in return for course credit (44 females; *M*_age_ = 20.0 years). All were native English speakers and had normal or corrected-to-normal vision.

#### Materials

The sentence context preceding the initial target for most of the 76 pairs of two-sentence passages were taken directly from Experiment 2.^5^ The average cloze probability of the related and unrelated unpredictable words was very low in both the strongly and weakly constraining contexts, but, importantly, very high for the predictable but never presented completions in the strongly constraining contexts, indicating that the unpredictable words disconfirmed a highly probable completion in these contexts. The other lexical characteristics of the target words for each condition did not differ from those of Experiment 2.

#### Apparatus

There were no changes in the apparatus from the previous experiments.

#### Procedure

The procedure was identical to the previous experiments except that the 152 passages were counterbalanced across two lists. Comprehension questions appeared after approximately 36% of the trials (mean accuracy = 94.2%).

### Results

Data handling was identical to the previous experiments. Trials were removed either due to track loss or blinks on the region of interest (6.0% of trials). Fixations on the initial target below 80 ms, first fixation durations above 800 ms, gaze durations above 1,200 ms, and total durations above 2,000 ms were also excluded (1.0% of trials). These exclusions left 8,201 initial target datapoints (93.0% of the data) for analysis. The average reading measures on the initial target for each condition are presented in Table 6.

**Table 6.** Mean (and standard deviation) reading measures on the initial target word for each condition in Experiment 3.

Reading measure	Strongly constraining context		Weakly constraining context	
	Related initial target	Unrelated initial target	Related initial target	Unrelated initial target
Skipping (%)	26 (7)	23 (7)	23 (8)	23 (7)
First fixation (ms)	219 (16)	218 (14)	215 (16)	210 (16)
Gaze (ms)	244 (18)	240 (20)	235 (22)	228 (17)
Total fixation (ms)	314 (42)	336 (35)	346 (39)	335 (36)
Regressions-out (%)	14 (6)	15 (6)	17 (6)	16 (6)
Regressions-in (%)	18 (7)	22 (7)	26 (7)	23 (8)

As in the previous experiments, (G)LMMs were used to test the fixed effect of constraint nested under initial target type, which returned estimates of the main effect of initial target type and the constraint effect separately for related and unrelated targets. Power analyses using the same procedure as the previous experiments demonstrated sufficient power to detect the constraint effect for unrelated initial targets on gaze duration (.84) based on the 9 ms effect size observed in Experiment 2. Criteria for the random-effects structures and significance thresholds were identical to the previous experiments. A summary of the statistical analyses for the initial target is presented in Table 7.

**Table 7.** Results for the nested linear mixed effects models for log-transformed fixation duration measures and generalised linear mixed effects models for fixation probability measures on the initial target word in Experiment 3.

Measure	Fixed effect	*b*	*SE*	*t*/*z*
Skipping	Intercept	***–*1.32**	**0.11**	***–*12.08**
	Initial target type	*–*0.10	0.05	*–*1.88
	Related target: Constraint effect	*–*0.14	0.10	*–*1.39
	Unrelated target: Constraint effect	*–*0.01	0.09	*–*0.06
First fixation	Intercept	**5.33**	**0.02**	**342.05**
	Initial target type	*–*0.01	0.01	*–*1.25
	Related target: Constraint effect	*–*0.02	0.02	*–*1.09
	Unrelated target: Constraint effect	***–*0.04**	**0.01**	***–*2.76**
Gaze	Intercept	**5.40**	**0.02**	**292.55**
	Initial target type	*–*0.02	0.01	*–*1.95
	Related target: Constraint effect	*–*0.03	0.02	*–*1.71
	Unrelated target: Constraint effect	***–*0.05**	**0.02**	***–*3.22**
Total fixation	Intercept	**5.66**	**0.03**	**189.96**
	Initial target type	0.02	0.01	1.38
	Related target: Constraint effect	**0.09**	**0.03**	**3.70**
	Unrelated target: Constraint effect	*–*0.00	0.02	*–*0.16
Regressions-out	Intercept	***–*1.90**	**0.12**	***–*16.08**
	Initial target type	0.04	0.06	0.60
	Related target: Constraint effect	**0.33**	**0.13**	**2.60**
	Unrelated target: Constraint effect	0.09	0.11	0.79
Regressions-in	Intercept	***–*1.39**	**0.10**	***–*13.55**
	Initial target type	0.03	0.06	0.53
	Related target: Constraint effect	**0.51**	**0.11**	**4.44**
	Unrelated target: Constraint effect	0.06	0.12	0.53

Significant effects are bolded.

#### Initial target

The main effect of initial target type was not significant on any reading measures (|*t*/*z*|s < 1.95). For related unpredictable targets, there was a facilitatory constraint effect on total time, regressions-out and regressions–in (|*t*/*z*|s > 2.60) because related unpredictable targets received shorter reading times and fewer regressions-out and -in when presented in strongly compared with weakly constraining contexts. For unrelated unpredictable targets, there was evidence of prediction error cost because the constraint effect was significant on first fixation and gaze duration (|*t*|s > 2.76) reflecting longer reading times for unrelated unpredictable targets under conditions of strong compared with weak constraint.

Thus, the current results were almost identical to that of Experiment 2 in that strongly constraining contexts yielded processing benefits for related unpredictable targets and processing costs for unrelated unpredictable targets, although there was no evidence that readers subsequently recovered from encountering unrelated unexpected input in place of a more expected completion.

### Discussion

This experiment aimed to replicate and confirm the novel evidence of prediction error cost on the initial target word observed in Experiment 2. Experiment 3 increased statistical power by removing the predictable condition, leading to an increased number of items in the critical unpredictable conditions.

The results revealed that, consistent with the previous experiments, at initial presentation, related unpredictable targets elicited processing benefits in strongly constraining contexts despite violating a more expected completion. This included shorter total reading times due to fewer regressions-out and -in compared to the same targets in weakly constraining contexts. Furthermore, consistent with Experiment 2, unrelated unpredictable targets elicited immediate processing costs on gaze duration, as well as on the earlier measure of first fixation duration, in strongly compared with weakly constraining contexts, although these effects were also relatively small (12 and 8 ms, respectively). Unlike Experiment 2, however, there was no evidence that readers necessarily recovered from these immediate prediction error costs because there was no subsequent benefit on later measures for these unexpected words.

This evidence of prediction error costs may appear somewhat contradictory given that readers’ predictions about the initial target were never explicitly confirmed in Experiment 3. It is, therefore, possible that the presence of related unpredictable completions was sufficient to confirm readers’ general semantic expectancies despite being different lexical entities. Another explanation is that readers generated predictions about upcoming text during online processing because this mechanism is inherently useful, even if repeatedly disconfirmed for one word in the passage, as any amount of relevant preactivation is beneficial for subsequent processing (see van Wonderen & Nieuwland, 2023; Zhang et al., 2019). Overall then, readers do appear to engage in predictive processing during online language comprehension, which can lead to small processing costs when their expectations turn out to be incorrect.

## General discussion

Previous investigations of whether readers make predictions about the full identity of upcoming words have focused on the extent to which there are processing consequences when readers encounter linguistic input that is incompatible with their expectations. However, eye-movement studies to date have revealed contradictory and generally elusive evidence of such prediction error costs for unexpected input, leading researchers to conclude that readers do not routinely predict or anticipate upcoming words during online processing (Andrews et al., 2022; Frisson et al., 2017; Luke & Christianson, 2016; Wong et al., 2022; but see Cevoli et al., 2022). This is despite evidence from ERP studies that unexpected input presented in place of a more expected completion elicits a late frontal positivity that has been linked to higher-order suppression and revision processes following initial semantic access (see Van Petten & Luka, 2012 for a review). The present eye-movement research used controlled experimental designs to examine whether readers’ lexical predictions are observable during the immediate processing of critical words. A further major novel contribution of this research was to use a repetition paradigm adapted from the study by Rommers and Federmeier (2018a, 2018b) to look for evidence of anticipatory prediction that may be observable downstream from the initial presentation of critical words, even if readers’ expectations are disconfirmed and replaced by less expected input.

### Are there immediate consequences of lexical prediction?

This research revealed that, at initial presentation, unexpected input that disconfirmed a more expected completion yielded immediate prediction error costs. Specifically, unpredictable completions that were semantically unrelated to the best completion disrupted readers’ eye movements in strongly constraining contexts on gaze duration in Experiment 2 when only plausible stimuli were included, and on first fixation and gaze duration in Experiment 3 when only unpredictable stimuli were included. This suggests that, across these experiments, readers were sensitive to a mismatch between the word expected in a constraining context and the input actually encountered, providing support for the idea that they do activate linguistic content and commit to their predictions ahead of time during online processing.

Notably, the consequences of misprediction experienced by the language processor in these two experiments were not severe or prolonged. First, the increase in processing times for unrelated unpredictable completions in strongly compared with weakly constraining contexts was 9 ms on gaze duration in Experiment 2; and 8 and 12 ms on first fixation and gaze duration, respectively, in Experiment 3. These effect sizes are relatively small, especially when compared to the average effect sizes of the processing benefits for predictable completions under conditions of strong compared with weak constraint reported in eye-movement studies (e.g., 16 and 24 ms on first fixation and gaze duration, respectively, Frisson et al., 2017), suggesting that the disrupted processing arising from incorrect predictions may be weaker than the typical facilitated processing accompanying correct predictions. Second, Experiment 2 revealed that although unrelated unpredictable completions yielded longer first-pass reading times under conditions of strong constraint, these items subsequently received shorter total reading times and fewer regressions-in, implying that readers were able to quickly resolve their disconfirmed predictions via integration processes that were supported by information extracted from the rest of the sentence. Supplementary analyses combining data from all three experiments confirmed that there was a significant overall constraint effect for unrelated unpredictable completions in the form of a cost on gaze duration and a benefit on regressions-in, indicating that, although the effects were small and short-lived, they do appear to be reliable and interpretable.^6^ Thus, although predictions about upcoming text lead to processing benefits when they turn out to be correct, the current findings suggest that the violation of these expectations leads to first-pass processing costs that are only small and short-lived for the language processor.

In contrast to Experiments 2 and 3, however, there was no evidence of processing costs for unexpected input in Experiment 1, which presented a subset of the same sentence frames. One explanation for the absence of prediction error costs in this experiment is that readers may have modulated the strength of their predictive processing in response to the noticeable proportion of anomalous completions in the broader linguistic environment. That is, when some of the stimuli were implausible (i.e., Experiment 1), the language processor may have been more likely to prioritise bottom-up input in the preceding context, allowing comprehension to unfold passively via lexical co-occurrence or spreading activation of associated concepts (Huettig, 2015). In contrast, when all of the stimuli were plausible (i.e., Experiments 2 and 3), the language processor may have been more likely to rely on top-down comprehension strategies including genuine lexical prediction because, as previously mentioned, this mechanism is inherently useful even if readers’ predictions are not always confirmed. Supplementary analyses confirmed that the magnitude of the predictability cost was attenuated in Experiment 1 compared to the subsequent two experiments on first fixation duration. Furthermore, target words in Experiment 1 received longer reading times on first-pass measures but fewer regressions-out and -in compared with target words in Experiments 2 and 3.^7^ As such, readers may have been sensitive to the linguistic incongruity in Experiment 1, which is consistent with previous research showing immediate effects of severe plausibility violations on readers’ processing strategies (Braze et al., 2002; Rayner et al., 2004; Veldre & Andrews, 2016; Veldre et al., 2020). However, this explanation for the discrepant findings across the experiments remains speculative and requires further systematic investigation to determine the precise impact of anomalies on predictive processing during online language comprehension.

Somewhat surprisingly, first-pass processing benefits for the most expected completion in strongly constraining contexts were also weaker than expected across the experiments. That is, despite evidence that incorrect predictions incur an immediate processing cost, the benefit of encountering a correct prediction was restricted to lower skipping rates and marginally shorter first fixation durations in Experiment 1, and to lower skipping rates only in Experiment 2, constrasting the robust first-pass predictability effects typically observed in previous eye-movement studies (Balota et al., 1985; Fitzsimmons & Drieghe, 2013; Rayner & Well, 1996; but see Calvo & Meseguer, 2002; Hyönä, 1993). As elaborated earlier, this is unlikely to be due to a weak manipulation of predictability given the large differences in cloze probability for the predictable target across the constraint conditions. However, it may be relevant that previous eye-movement studies have typically assessed predictability effects by comparing predictable and unpredictable targets within the same sentence context (Balota et al., 1985; Fitzsimmons & Drieghe, 2013) rather than by comparing the same predictable completion in strongly and weakly constraining contexts like in this study. Nonetheless, the fact that these predictability effects generally emerged on first-pass reading measures is consistent with the findings of previous eye-movement studies, which show that generating lexical predictions ahead of time affects the earliest stages of processing (Fitzsimmons & Drieghe, 2013; Frisson et al., 2017; Rayner et al., 2011; Rayner & Well, 1996).

Evidence of immediate prediction error costs for unexpected input, however, did not extend to unpredictable completions that were semantically related to the best completion of the strongly constraining contexts. Instead, consistent with previous eye-movement (Frisson et al., 2017; Luke & Christianson, 2016; Wong et al., 2022) and ERP investigations (Federmeier et al., 2002; Federmeier & Kutas, 1999; Thornhill & Van Petten, 2012), related unpredictable completions received facilitated processing in strongly compared with weakly constraining contexts across all three experiments, suggesting that these items may have been partially preactivated either due to spreading activation from the most expected completion (Neely, 1977) or because the context independently activated a set of plausible continuations based on the available semantic information (Luke & Christianson, 2016; Roland et al., 2012). These facilitatory effects, however, did not affect the early measures on which predictability effects are typically observed (Balota et al., 1985; Fitzsimmons & Drieghe, 2013; Rayner & Well, 1996). Instead, they were restricted to the late measures of total reading time and regressions-in, implying that related unpredictable completions were easier to integrate into the unfolding discourse representation. The apparent immediate cost of encountering unexpected input therefore appears to be mitigated entirely by semantic similarity with the disconfirmed prediction, which instead serves to facilitate subsequent integration processes.

The current findings are therefore consistent with the study by Cevoli et al. (2022)—the only eye-movement study to date to demonstrate that readers are sensitive to the costs of making an incorrect prediction. It should be noted, however, that the findings by Cevoli et al. were based on the Provo corpus (Luke & Christianson, 2017), which shows no evidence of such costs when analysed via the cloze metric of predictability (Andrews et al., 2022; Luke & Christianson, 2016). Moreover, several key aspects of the study by Cevoli et al. differ from this study including their use of surprisal and entropy derived from a language model to estimate word predictability and their use of corpus data, which allows predictable and unpredictable words to vary on multiple uncontrolled dimensions. Thus, this research using controlled experimental designs arguably provides more compelling evidence that readers are capable of using their prior knowledge and experiences about the context and the world to generate predictions about upcoming text. This evidence of predictive processing during real-time language comprehension is in line with broader predictive accounts of cognitive functioning (Clark, 2013; Friston, 2010).

The current findings, however, do differ from two previous eye-movement studies investigating prediction error costs using similar controlled experimental designs (Frisson et al., 2017; Wong et al., 2022). Before speculating on some potential explanations for these discrepant findings, it is necessary to consider several factors that may have contributed to the longer processing times on unrelated unpredictable completions in strongly constraining contexts observed in Experiments 2 and 3. One possibility that can be ruled out is differences in the lexical characteristics of unrelated unpredictable completions, such as their frequency and length. Because the current experiments always presented the same targets in strongly and weakly constraining contexts, lexical characteristics were perfectly matched across the two context conditions and therefore could not have contributed to the observed evidence of prediction error costs. Another possibility is that the evidence of prediction error costs reflects the slightly lower plausibility ratings for unrelated unpredictable completions in strongly compared with weakly constraining contexts (4.2 vs 4.6 out of 5). However, this average difference in plausibility is negligible especially in comparison with previous eye-movement studies of plausibility effects in which these differences are much larger (e.g., 1–2 vs 4–5 based on a five-point scale; Rayner et al., 2004; Staub et al., 2007; Veldre et al., 2020).^8^ Predictability and plausibility are generally correlated (see Nieuwland et al., 2020) and, as such, it can be difficult to disentangle their relative contributions during online and offline language processing—readers are inherently more likely to rate an unpredictable word that is semantically unrelated to their expectations as implausible even if it is an acceptable continuation (see also Frisson et al., 2017). Thus, to the extent that effects of plausibility are separable from predictability, the small differences in rated plausibility between unrelated unpredictable completions in strongly and weakly constraining contexts are also unlikely to be responsible for the evidence of prediction error costs observed.

The current findings may have differed from that of previous eye-movement studies for several reasons. First, Frisson et al. (2017) who presented identical experimental conditions to Experiment 2 observed no evidence of processing costs for unexpected input in strongly constraining contexts. One possible reason for this discrepancy is that our materials had a stronger predictability manipulation—the average cloze probability of the predictable targets under conditions of strong constraint were slightly higher in our experiments that revealed evidence of prediction error costs (.81 and .83, respectively) compared with the experiment by Frisson et al. that did not (.77). More generally, our experiments had higher statistical power—compared with 20 items per condition in the experiment by Frisson et al., participants read ~25 items per condition in Experiment 2, and 38 items per condition in Experiment 3. Evidence of prediction error costs in the eye-movement record therefore appears to depend not only on the strength of readers’ predictive processing during reading, but also on an adequately powered experimental design to detect the relatively small and short-lived effects on early fixation duration measures.

Second, Wong et al. (2022) who presented three-sentence passages that varied in their source of constraint violation also found no evidence of processing costs for unexpected input in either globally or locally constraining contexts. It is possible that the extended contexts used in the study by Wong et al. provided readers with more time and opportunity to passively activate general morphosynactic, syntactic, and semantic information about upcoming words as part of the natural reading process. Because this would have likely led to the partial preactivation of multiple lexical candidates including those of low cloze probability, unexpected input may have been less likely to lead to processing costs when eventually encountered. In contrast, the minimal contexts used in this study may have provided readers with less time and opportunity for similar activation of such information. Given that fewer low cloze continuations would have been partially preactivated ahead of time, unexpected input may have been more likely to lead to processing costs when eventually encountered, as observed in the current experiments.

More generally, another explanation for the current findings is simply that readers did activate and commit to a single lexical candidate ahead of time because of specific stimulus and participant characteristics. Predictions that turned out to be incorrect therefore led to processing costs, even if these consequences were small and short-lived for the language processor. Indeed, there is growing evidence to suggest that readers’ use of predictive processing is determined to a variety of factors that go beyond the linguistic content presented (see Huettig & Mani, 2016 for a review). For example, certain task demands and goals have been shown to increase the extent to which readers rely on lexical prediction, including explicit instructions to predict passage-final words and report the accuracy of these predictions (Brothers et al., 2015, 2017; Dave et al., 2018), and tasks that involve proofreading for semantically incongruent words compared with reading for comprehension (Andrews et al., 2022; Schotter et al., 2014). As such, readers in the current experiments that revealed evidence of prediction error costs may have also generated stronger lexical predictions about upcoming text because, for reasons that remain unclear, it was beneficial or necessary for their current task demands and goals (see also Federmeier, 2021; Pickering & Gambi, 2018). However, further research is clearly necessary to clarify the factors that influence whether and how anticipatory prediction unfolds during real-time language comprehension.

### Are there downstream consequences of lexical prediction?

The major novel question of this research was whether evidence of anticipatory prediction was also observable downstream from the initial point of activation and, more specifically, when readers’ expectations were disconfirmed and replaced by less expected input. Thus, after each initial sentence, a thematically related unconstraining sentence was presented to probe the downstream activation of the previously predictable completion as a function of whether it had been confirmed or disconfirmed (Experiments 1 and 2). Downstream targets that were repeated words, because a predictable completion was previously encountered, were processed more efficiently than downstream targets that were new words, because an unpredictable completion was previously encountered, but this was only observed in Experiment 2 and not in Experiment 1, which included anomalous stimuli. This finding suggests that, consistent with previous observations across different methodologies (Hyönä & Niemi, 1990; Kamienkowski et al., 2018; Raney & Rayner, 1995 using eye-tracking; Scarborough et al., 1977 using lexical decision; Lowder et al., 2013; Masson & Freedman, 1990 using naming; Van Petten et al., 1991 using ERPs), readers generally benefitted from seeing a word more than once, and that, more importantly, there were observable downstream consequences of processing the initial sentence. However, this repetition benefit may have been sensitive to the broader linguistic environment when it contained a noticeable proportion of implausible stimuli, providing further evidence that readers’ normal reading strategies were disrupted by the presence of linguistic incongruity (Braze et al., 2002; Rayner et al., 2004; Veldre & Andrews, 2016; Veldre et al., 2020).

Across both experiments, however, there was no evidence that the repetition benefit was modulated by whether previously predictable completions were presented in strongly compared with weakly constraining contexts. This is inconsistent with the ERP study by Rommers and Federmeier (2018b), which reported an attenuated repetition benefit for previously predictable completions, suggesting that these items had been encoded less thoroughly during their initial presentation, most likely because the language processor was simply verifying what the context already supported (Hubbard et al., 2019; Rommers & Federmeier, 2018b; Van Berkum, 2010). Although the current findings provide no evidence to support this account, the null effects also do not provide evidence to support the opposite of this account, i.e., more thorough encoding of previously predictable completions, which would be expected to free up more of readers’ cognitive resources and lead to a stronger repetition benefit. One methodological factor that could have interfered with these effects is the fact that participants in the current experiments always read both strongly and weakly constraining versions of each item. As such, they may have encountered the critical downstream target on a previous trial, which could have contributed to the weaker than expected repetition effects observed, regardless of whether the downstream target had been previously predictable or not. Further systematic investigations are therefore necessary to clarify how thoroughly predictable completions are processed during their initial presentation.^9^

Furthermore, downstream targets that were new words also received processing facilitation following their initial presentation across both experiments. This was observed most consistently when following related compared with unrelated initial targets, providing further evidence of downstream processing consequences when initial and downstream targets were semantically similar. However, downstream new targets were minimally affected by the presence of a previously predictable, but never presented, word. This is inconsistent with previous ERP studies that reported pseudo-repetition effects for readers’ lexical predictions even if they are merely expected but never presented (Rommers & Federmeier, 2018a). The only evidence that disconfirmed predictions had downstream consequences was following the presence of anomalous unpredictable completions in Experiment 1, which led to higher skipping rates for new downstream words. These facilitatory downstream effects, however, were restricted to a single eye-movement measure and were not replicated in the subsequent experiment that revealed readers’ immediate sensitivity to failed predictions. Thus, the overall picture that emerges is that, in contrast to the ERP literature (Hubbard et al., 2019; Lai et al., 2021; Rommers & Federmeier, 2018a, 2018b), the current findings provide no consistent evidence that effects of anticipatory prediction are observable downstream from their initial presentation.

One possible explanation for this discrepancy is that the impact of anticipatory prediction during online processing is genuinely short-lived or at least only observable during the immediate processing of critical words in the eye-movement record. However, the impact of anticipatory prediction may have been enhanced in ERP studies because of the stimuli presentation format. Because ERP studies typically present sentence stimuli word-by-word for a fixed duration that not only increases the processing time for each word (Degno & Liversedge, 2020; Rayner, 2009; Rayner & Clifton, 2009) but also precludes readers’ ability to skip words, re-read previous parts of text, and use upcoming parafoveal information, readers could be implicitly encouraged to rely on predictive processes more than would be expected during normal reading (Dambacher et al., 2012; Wlotko & Federmeier, 2015). Such a possibility would account for the consistent ERP evidence of immediate and downstream effects of disconfirmed predictions.

Furthermore, some aspects specific to the study by Rommers and Federmeier (2018a) could have contributed to the increased impact of lexical prediction on readers’ downstream processing. Participants in this study were presented with sequences of unrelated sentences in which readers’ expectations were confirmed or disconfirmed several sentences before the critical target was presented to probe their downstream representation. Because the inclusion of unrelated, intervening sentences could have prevented the construction of a coherent discourse representation especially when comprehension probes were not included throughout the task, readers may have been less likely to suppress and revise their prior expectations following unexpected input beyond the first sentence in which it appeared, leading to the facilitatory downstream effects observed. Thus, it appears important for future research to clarify the extent to which these methodological factors are responsible for the downstream consequences of lexical prediction observed in ERP studies.

In summary, the present findings provide some of the first eye-movement evidence within a controlled experimental design of readers making predictions about the precise lexical identity of upcoming words in advance of their presentation. The consequences of making an incorrect prediction, however, appeared to be small and short-lived and observable only during the immediate, and not downstream, processing of unexpected input. Notably, these immediate prediction error costs only emerged in plausible linguistic environments, suggesting that readers strategically modulated their predictive processing based on information in their broader linguistic environment. This extends previous observations that predictive processes can depend on a variety of factors including age, individual differences, and task and goal demands (see Huettig, 2015). Taken together, although predictive processes can serve to facilitate language processing, its usage may not be as automatic or ubiquitous as previously assumed.

## Supplemental Material

sj-docx-1-qjp-10.1177_17470218231223858 – Supplemental material for Looking for immediate and downstream evidence of lexical prediction in eye movements during reading Supplemental material, sj-docx-1-qjp-10.1177_17470218231223858 for Looking for immediate and downstream evidence of lexical prediction in eye movements during reading by Roslyn Wong, Aaron Veldre and Sally Andrews in Quarterly Journal of Experimental Psychology

## Notes

1. The responses to eight comprehension questions were removed as less than 66% of participants answered them correctly. The original comprehension accuracy was 86.3%. These eight comprehension questions were rewritten for the subsequent two experiments.

2. Eye-tracking participants also provided cloze completions after a 1–2-week delay to obtain individualised estimates of cloze probability for the target words (*n* = 57 as two participants did not return). These individualised cloze data were virtually identical to the cloze data collected from the independent sample of participants and are therefore not reported. Based on these individualised cloze data, a separate set of (G)LMMs were conducted excluding: (a) trials where participants did not generate the predictable initial target in the strongly constraining contexts and (b) trials where participants generated the unpredictable initial target in the weakly constraining contexts (9.9% of trials). The pattern of significant results of these restricted analyses was identical to the unrestricted analyses with the following exceptions: for the initial target, the relatedness effect was significant on regressions-in (*z* = –2.69); for anomalous initial targets, the constraint effect was not significant on first fixation duration (*t* = –1.70) or regressions-in (*z* = 1.94).

3. The predictability benefit (i.e., the more efficient processing for predictable words in strongly compared with weakly constraining contexts) on the probability of regressions-out was 1% in Experiment 1 compared with 5% in Experiment 2 (*b* = 0.42, *SE* = 0.19, *z* = 2.24). The predictability cost (i.e., the less efficient processing for unrelated unpredictable words in strongly compared with weakly constraining contexts) on first fixation duration was –3 ms in Experiment 1 compared with 8 ms in Experiment 2 (*b* = –0.05, *SE* = 0.02, *t* = –2.30).

4. For target words in Experiment 1 (excluding the anomalous conditions) compared with target words in Experiment 2, total fixation durations were 326 ms (*SD* = 28) and 368 ms (*SD* = 37), respectively (*b* = 0.09, *SE* = 0.04, *t* = 2.26), regressions-out were 11% (*SD* = 1) and 15% (*SD* = 2), respectively (*b* = 0.27, *SE* = 0.13, *z* = 2.03), and regressions-in were 18% (*SD* = 7) and 27% (*SD* = 8), respectively (*b* = 0.65, *SE* = 0.13, *z* = 5.03). Experiment 1 target words also showed numerically fewer skips (25% vs 28%) and longer reading times on first-pass measures (first fixation duration: 226 vs 216 ms; gaze duration: 249 vs 237 ms) than Experiment 2 target words.

5. Cloze completions for eight modified items were collected from a separate sample of 19 participants (12 females; *M*_age_ = 18.9 years).

6. For unrelated unpredictable completions in strongly compared with weakly constraining contexts averaged across the three experiments (*N* = 177), gaze duration was 246 ms (*SD* = 7) and 238 ms (*SD* = 12), respectively, (*b* = –0.03, *SE* = 0.01, *t* = –2.53) and the probability of regressions-in was 22% (*SD* = 5) vs 28% (*SD* = 6), respectively, (*b* = 0.32, *SE* = 0.10, *t* = 3.33).

7. The predictability cost (i.e., the less efficient processing for unrelated unpredictable words in strongly compared with weakly constraining contexts) on first fixation duration was –3 ms in Experiment 1 compared with 8 ms in Experiments 2 and 3 (*b* = 0.05, *SE* = 0.02, *t* = 2.87). For (related and unrelated) target words in Experiment 1 compared with Experiments 2 and 3, first fixation durations were 230 ms (*SD* = 2) and 217 ms (*SD* = 4), respectively (*b* = 0.05, *SE* = 0.02, *t* = 2.48), gaze durations were 254 ms (*SD* = 3) and 240 ms (*SD* = 7), respectively (*b* = 0.06, *SE* = 0.02, *t* = 2.39), regressions-out were 12% (*SD* = 1) vs 15% (*SD* = 1), respectively (*b* = –0.31, *SE* = 0.13, *z* = –2.47), and regressions-in were 20% (*SD* = 6) vs 26% (*SD* = 6), respectively (*b* = –0.35, *SE* = 0.12, *z* = –3.03).

8. Supplementary analyses addressed this potential confound by removing 24 items that were rated 3.5 or lower in plausibility, i.e., leaving 52 items rated above the mid-point of the scale. The plausibility ratings for unrelated unpredictable targets in strongly and weakly constraining contexts in this subset of items were 4.5 (*SD* = 0.4) and 4.8 (*SD* = 0.3), respectively. Evidence of prediction error costs on gaze duration remained in the expected direction in Experiment 2, i.e., significantly longer processing times for unrelated unpredictable completions in strongly (*M* = 242 ms, *SD* = 32) compared with weakly constraining contexts (*M* = 230 ms, *SD* = 31; *b* = –0.05, *SE* = 0.02, *t* = –2.08). Although evidence of prediction error costs on first-pass measures was not significant in Experiment 3, the numerical trend was in the expected direction, i.e., longer processing times for unrelated unpredictable completions in strongly (first fixation duration: *M* = 217 ms, *SD* = 16; gaze duration: *M* = 238 ms, *SD* = 24) compared with weakly constraining contexts (first fixation duration: *M* = 213 ms, *SD* = 19; gaze duration: *M* = 233 ms, *SD* = 22).

9. Even if facilitatory downstream effects were observed following previously predictable completions in strongly compared with weakly constraining contexts, the paradigm by Rommers and Federmeier (2018a) does not make it possible to determine whether this facilitation was due to the prior prediction per se, due to the initial context that led to this prediction, or due to a combination of both factors.
