# Supplementary material for: Looking for immediate and downstream evidence of lexical prediction in eye movements during reading
Source: Q J Exp Psychol (Hove). 2024 Jan 27;77(10):2040–64. doi: 10.1177/17470218231223858 (PMC11453035; doi:10.1177/17470218231223858)
Supplement: sj-docx-1-qjp-10.1177_17470218231223858 – Supplemental material for Looking for immediate and downstream evidence of lexical prediction in eye movements during reading [file sj-docx-1-qjp-10.1177_17470218231223858.docx]

Supplementary Material for:

**Looking for Immediate and Downstream Evidence of Lexical Prediction in Eye Movements during Reading**

Roslyn Wong^1^, Aaron Veldre^1,2^, & Sally Andrews^1^

School of Psychology, The University of Sydney

School of Psychological Sciences, Macquarie University

**Author Note**

In memoriam, we thank Sally Andrews for her contributions to this research and to scientific studies of reading.

Roslyn Wong is now at Macquarie University.

This research was supported under Australian Research Council’s *Discovery Projects* funding scheme (project numbers DP18102705, DP190100719) and The University of Sydney’s Postgraduate Research Support Scheme.

Portions of these data were presented at the 60^th^ Annual Meeting of the Psychonomic Society. The experimental materials, data, and analysis code from the present study are publicly available at the Open Science Framework website: <https://osf.io/5rgck/>. This study was not preregistered.

Correspondence concerning this article should be addressed to Roslyn Wong, School of Psychological Sciences, Macquarie University, NSW, 2109, Australia. Email: [roslyn.wong@mq.edu.au](mailto:roslyn.wong@mq.edu.au)

**Analysis of Downstream Target in Experiment 3**

The downstream target in Experiment 3 was either the predictable, but never presented, completion from the strongly constraining sentence or the other plausible, unpredictable completion (see Table S1 for an example item pair).

Data handling was identical to the experiments reported in the main paper. Trials were removed either due to track loss or blinks on the region of interest (2.2% of trials). Fixations on the downstream targets below 80ms, first fixation durations above 800 ms, gaze durations above 1200 ms, and total durations above 2000 ms were also excluded (1.7% of trials). These exclusions left 8473 downstream target datapoints (96.1% of the data) for analysis. The average reading measures on the downstream target for each condition are presented in Table S2.

As in the previous experiments, (G)LMMs tested the fixed effect of constraint nested under condition which returned estimates of the main effect of condition and the constraint effect separately for the four types of downstream targets (previously predictable downstream targets following related initial targets, previously unrelated downstream words following related initial targets, previously predictable downstream words following unrelated initial targets, and previously related downstream words following unrelated initial targets). Condition was coded as a set of three orthogonal contrasts which tested: (1) the initial target type effect – the difference between the related and unrelated initial target conditions, (2) the downstream effect following related initial targets – the difference between the previously predictable and unrelated downstream words following related initial target conditions, and (3) the downstream effect following unrelated initial targets – the difference between the previously predictable and related downstream words following unrelated initial target conditions. Criteria for the maximal random-effects structures and significance thresholds were identical to the previous experiments. A summary of the statistical analyses for the downstream target is presented in Table S3.

The initial target type effect was not significant on any reading measures at the downstream target (|*t*/*z|*s<1.29). The downstream effect following related targets was significant on gaze duration, total fixation duration, and regressions-in (|*t*/*z|*s>3.08) – downstream targets following related initial words received shorter reading times and fewer regressions-in when they were the previously predictable versus unrelated word. The downstream effect following unrelated targets was significant on total fixation duration and regressions-in (*t*/*z|*s>4.08) – downstream targets following unrelated initial words received shorter reading times and fewer regressions-in when they were the previously predictable versus related word. The effect of *constraint* was not significant on any of the reading measures at the downstream target regardless of the completion that appeared in the first sentence (|*t*/*z*|s<1.47). Thus, consistent with the previous experiments, there was no indication that readers’ lexical predictions were observable downstream from their initial point of activation.

**Table S1**

*Example set of items and mean (and standard deviation) stimulus characteristics in Experiment 3*

|  |  | Cloze probability | |
| --- | --- | --- | --- |
| Condition | Example item  (Initial target bolded; Downstream target underlined) | Initial target | Expected word |
| Strongly constraining context | |  |  |
| Related initial target, Previously predictable downstream target | Irene and her husband travelled by boat to the tropical **resort** for their honeymoon. It was close to the island they had chosen for their wedding | .01 (.03) | .83 (.13) |
| Related initial target, Previously unrelated downstream target | Irene and her husband travelled by boat to the tropical **resort** for their honeymoon. It was close to the garden they had chosen for their wedding | .01 (.03) | .83 (.13) |
| Unrelated initial target, Previously predictable downstream target | Irene and her husband travelled by boat to the tropical **garden** for their honeymoon. It was close to the island they had chosen for their wedding | .00 (.02) | .83 (.13) |
| Unrelated initial target, Previously related downstream target | Irene and her husband travelled by boat to the tropical **garden** for their honeymoon. It was close to the resort they had chosen for their wedding | .00 (.02) | .83 (.13) |
|  |  |  |  |
| Weakly constraining context | |  |  |
| Related initial target, Previously predictable downstream target | Today we visited a beautiful **resort** full of exotic birds. Tomorrow we will go to the island where we will spend the last few days of our holiday. | .01 (.03) | .01 (.03) |
| Related initial target, Previously unrelated downstream target | Today we visited a beautiful **resort** full of exotic birds. Tomorrow we will go to the garden where we will spend the last few days of our holiday. | .01 (.03) | .01 (.03) |
| Unrelated initial target, Previously predictable downstream target | Today we visited a beautiful **garden** full of exotic birds. Tomorrow we will go to the island where we will spend the last few days of our holiday. | .01 (.05) | .01 (.03) |
| Unrelated initial target, Previously related downstream target | Today we visited a beautiful **garden** full of exotic birds. Tomorrow we will go to the resort where we will spend the last few days of our holiday. | .01 (.05) | .01 (.03) |

**Table S2**

*Mean (and standard deviation) reading measures on the downstream target word for each condition in Experiment 3*

|  | Strongly constraining context | | | | Weakly constraining context | | | |
| --- | --- | --- | --- | --- | --- | --- | --- | --- |
| Reading measure | Related initial target-Previously predictable downstream target | Related initial target-Previously unrelated downstream target | Unrelated initial target-Previously predictable downstream target | Unrelated initial target-Previously related downstream target | Related initial target-Previously predictable downstream target | Related initial target-Previously unrelated downstream target | Unrelated initial target-Previously predictable downstream target | Unrelated initial target-Previously related downstream target |
| Skipping (%) | 28 (12) | 26 (10) | 28 (12) | 27 (11) | 29 (10) | 28 (10) | 25 (9) | 27 (10) |
| First fixation | 192 (18) | 193 (17) | 192 (16) | 195 (16) | 189 (19) | 193 (18) | 191 (18) | 190 (18) |
| Gaze | 206 (24) | 212 (24) | 207 (22) | 212 (24) | 203 (21) | 215 (24) | 212 (22) | 213 (28) |
| Total fixation | 282 (42) | 287 (32) | 270 (41) | 298 (38) | 270 (39) | 300 (39) | 286 (31) | 299 (38) |
| Regressions-out (%) | 25 (11) | 27 (11) | 25 (10) | 25 (12) | 26 (10) | 27 (10) | 26 (9) | 28 (9) |
| Regressions-in (%) | 19 (8) | 23 (11) | 17 (9) | 23 (10) | 19 (10) | 24 (9) | 19 (10) | 22 (9) |

**Table S3**

*Results for the nested linear mixed effects models for log-transformed fixation duration measures and generalized linear mixed effects models for fixation probability measures on the downstream target word in Experiment 3. Significant effects are bolded.*

| Measure | Fixed effect | *b* | *SE* | *t*/*z* |
| --- | --- | --- | --- | --- |
| Skipping | **Intercept** | **-1.09** | **0.10** | **-11.09** |
|  | Initial target type | 0.05 | 0.05 | 1.02 |
|  | Related initial target: Downstream effect | 0.12 | 0.07 | 1.67 |
|  | Unrelated initial target: Downstream effect | -0.02 | 0.07 | -0.29 |
|  | Related initial target-Previously Predictable downstream: Constraint effect | 0.02 | 0.13 | 0.12 |
|  | Related initial target-Previously Unrelated downstream: Constraint effect | 0.13 | 0.15 | 0.88 |
|  | Unrelated initial target-Previously Predictable downstream: Constraint effect | -0.13 | 0.13 | -1.07 |
|  | Unrelated initial target-Previously Related downstream: Constraint effect | 0.02 | 0.12 | 0.13 |
|  |  |  |  |  |
| First fixation | **Intercept** | **5.19** | **0.02** | **301.24** |
|  | Initial target type | 0.00 | 0.01 | 0.21 |
|  | Related initial target: Downstream effect | -0.02 | 0.01 | -1.56 |
|  | Unrelated initial target: Downstream effect | 0.00 | 0.01 | 0.02 |
|  | Related initial target-Previously Predictable downstream: Constraint effect | -0.03 | 0.03 | -1.30 |
|  | Related initial target-Previously Unrelated downstream: Constraint effect | -0.00 | 0.02 | -0.20 |
|  | Unrelated initial target-Previously Predictable downstream: Constraint effect | -0.01 | 0.02 | -0.38 |
|  | Unrelated initial target-Previously Related downstream: Constraint effect | -0.02 | 0.02 | -0.81 |
|  |  |  |  |  |
| Gaze | **Intercept** | **5.27** | **0.02** | **279.85** |
|  | Initial target type | -0.00 | 0.01 | -0.50 |
|  | **Related initial target: Downstream effect** | **-0.04** | **0.01** | **-3.08** |
|  | Unrelated initial target: Downstream effect | -0.00 | 0.01 | -0.29 |
|  | Related initial target-Previously Predictable downstream: Constraint effect | -0.02 | 0.03 | -0.64 |
|  | Related initial target-Previously Unrelated downstream: Constraint effect | 0.01 | 0.03 | 0.46 |
|  | Unrelated initial target-Previously Predictable downstream: Constraint effect | 0.02 | 0.03 | 0.55 |
|  | Unrelated initial target-Previously Related downstream: Constraint effect | -0.00 | 0.03 | -0.01 |
|  |  |  |  |  |
| Total fixation | **Intercept** | **5.52** | **0.02** | **226.17** |
|  | Initial target type | -0.01 | 0.01 | -0.97 |
|  | **Related initial target: Downstream effect** | **-0.07** | **0.02** | **-4.23** |
|  | **Unrelated initial target: Downstream effect** | **-0.06** | **0.02** | **-4.08** |
|  | Related initial target-Previously Predictable downstream: Constraint effect | -0.04 | 0.03 | -1.28 |
|  | Related initial target-Previously Unrelated downstream: Constraint effect | 0.03 | 0.03 | 1.18 |
|  | Unrelated initial target-Previously Predictable downstream: Constraint effect | 0.05 | 0.03 | 1.47 |
|  | Unrelated initial target-Previously Related downstream: Constraint effect | 0.00 | 0.03 | 0.12 |
|  |  |  |  |  |
| Regressions-out | **Intercept** | **-1.19** | **0.10** | **-11.96** |
|  | Initial target type | 0.02 | 0.05 | 0.31 |
|  | Related initial target: Downstream effect | -0.06 | 0.08 | -0.85 |
|  | Unrelated initial target: Downstream effect | -0.09 | 0.08 | -1.13 |
|  | Related initial target-Previously Predictable downstream: Constraint effect | 0.09 | 0.18 | 0.52 |
|  | Related initial target-Previously Unrelated downstream: Constraint effect | -0.05 | 0.18 | -0.29 |
|  | Unrelated initial target-Previously Predictable downstream: Constraint effect | 0.08 | 0.18 | 0.44 |
|  | Unrelated initial target-Previously Related downstream: Constraint effect | 0.14 | 0.18 | 0.76 |
|  |  |  |  |  |
| Regressions-in | **Intercept** | **-1.56** | **0.12** | **-13.48** |
|  | Initial target type | 0.08 | 0.06 | 1.29 |
|  | **Related initial target: Downstream effect** | **-0.35** | **0.08** | **-4.29** |
|  | **Unrelated initial target: Downstream effect** | **-0.34** | **0.08** | **-4.09** |
|  | Related initial target-Previously Predictable downstream: Constraint effect | -0.02 | 0.20 | -0.13 |
|  | Related initial target-Previously Unrelated downstream: Constraint effect | 0.04 | 0.16 | 0.28 |
|  | Unrelated initial target-Previously Predictable downstream: Constraint effect | 0.15 | 0.18 | 0.84 |
|  | Unrelated initial target-Previously Related downstream: Constraint effect | -0.12 | 0.17 | -0.72 |
